# Molecular Epidemiology and Conjugative Transferability of *bla*_OXA-48_/*bla*_NDM-1_ Co-Occurrence in Extensively Drug-Resistant *Klebsiella pneumoniae* from Egyptian Tertiary Care Hospitals

**DOI:** 10.3390/pathogens15101043

**Published:** 2026-10-02

**Authors:** Mohamed Abd El-Gawad El-Sayed Ahmed, Amr Elsayed Ibrahim Elghopashy, Yasser M. Ragab, Reham R. EL-Lakany, Doaa Abdelrahman, Alaa A. Omar, Reham M. Goda

**Affiliations:** 1 Department of Microbiology and Immunology, College of Pharmaceutical Sciences and Drug Manufacturing, Misr University for Science and Technology, Giza P.O. Box 77, Egypt; amr.elghopashy@must.edu.eg; 2 Department of Microbiology and Immunology, Faculty of Pharmacy, Cairo University, Cairo 11562, Egypt; 3 Department of Microbiology and Immunology, Faculty of Pharmacy, Sinai University, Al-Arish 45518, Egypt; 4 Department of Microbiology and Immunology, Faculty of Pharmacy, Horus University, Damietta 34518, Egypt; 5 Department of Internal Medicine, College of Medicine, Princess Nourah Bint Abdulrahman University, P.O. Box 84428, Riyadh 11671, Saudi Arabia; dsabdelrahman@pnu.edu.sa; 6 Nanomedicine Research Unit, Delta University for Science and Technology, Gamasa 11152, Egypt; alaa.bokharist@deltauniv.edu.eg; 7 Department of Microbiology and Immunology, Faculty of Pharmacy, Delta University for Science and Technology, Gamasa 11152, Egypt; reham.gouda@deltauniv.edu.eg

**Keywords:** *Klebsiella pneumoniae*, carbapenemases, multidrug resistance, extensively drug-resistant bacteria, plasmid conjugation, *bla*_OXA-48_, *bla*_NDM-1_, Egypt, sequencing

## Abstract

Carbapenem-resistant *Klebsiella pneumoniae* is a major threat to global health, with increasing incidence in developing countries, including Egypt. This study determined carbapenem-resistance prevalence among clinical *K. pneumoniae* isolates from two Egyptian tertiary hospitals, characterized the major carbapenemase genes, and assessed the transferability of the resistance plasmids. VITEK^®^ 2 and MALDI-TOF MS identified 94 non-duplicate isolates. Susceptibility was determined by Kirby–Bauer diffusion and VITEK^®^ 2. MICs of imipenem, meropenem and colistin were confirmed by broth microdilution. Isolates were classified as multidrug-resistant (MDR), extensively drug-resistant (XDR), or pandrug-resistant (PDR) according to established definitions. Phenotypic (modified Hodge test [MHT], modified carbapenem inactivation method [mCIM], modified Carba NP test [mCNP]) and genotypic (PCR/BlastN) evaluation of carbapenemase production and plasmid transferability by conjugation were carried out in 18 isolates, selected to represent every carbapenemase genotype identified in the collection. All isolates were MDR, and 94.68% were carbapenem-resistant and fulfilled the criteria for XDR, remaining susceptible only to colistin and tigecycline. Carbapenemase production was confirmed phenotypically in 98.9% and genotypically in 97.9% of isolates. *bla*_OXA-48_ (94.7%) and *bla*_NDM-1_ (69.1%) predominated, with 67.0% co-carriage; *bla*_KPC_ occurred in 5.3%. Conjugation transferred carbapenemase-encoding plasmids to *Escherichia coli* J53 from 77.8% (14/18) of donors, conferring carbapenem non-susceptibility in all transconjugants. All isolates were colistin-susceptible by broth microdilution, the reference method. The high XDR prevalence, along with the conjugative transferability of *bla*_OXA-48_/*bla*_NDM-1_, is consistent with a mobile carbapenemase-encoding plasmid reservoir in Egyptian tertiary care, highlighting the significance of carbapenemase surveillance and stewardship. Plasmid characterization and clonal typing were not performed and are required to define that reservoir fully.

## 1. Introduction

*Klebsiella pneumoniae* is a Gram-negative opportunistic ESKAPE pathogen that causes serious healthcare-associated infections such as pneumonia, septicemia, meningitis, and urinary tract infections, especially in critically ill and immunosuppressed patients [1,2]. Beyond classical, hospital-associated strains, an increasing number of reports describe hypervirulent *K. pneumoniae* lineages that combine an expanded virulence-factor repertoire, including capsular, lipopolysaccharide, fimbrial and siderophore-mediated iron-acquisition mechanisms, with the capacity to cause severe, community-acquired disease in previously healthy individuals, underscoring the clinical importance of characterizing both the resistance and the virulence dimensions of this pathogen [3,4]. *K. pneumoniae* acquires β-lactamase-encoding genes, particularly extended-spectrum β-lactamases (ESBLs) and later carbapenemases, by taking up mobile genetic elements, mainly plasmids and transposons, shifting from multidrug resistance (MDR) to extensively drug-resistant (XDR) and pandrug-resistant (PDR) phenotypes [1,5,6]. Carbapenems were historically the last resort agents for ESBL- and AmpC-producing Enterobacteriaceae, but the worldwide emergence of carbapenem-resistant Enterobacteriaceae (CRE) and, in particular, carbapenem-resistant *K. pneumoniae* (CR-KP), has diminished this therapeutic reserve [7,8]. Now distributed across low-, middle- and high-income countries [9], CR-KP has been categorized by the World Health Organization as a critical-priority pathogen for new antibiotic development [10] and is associated with mortality rates of up to 50% in bloodstream infections [9]. Resistance arises through three major mechanisms: reduced outer-membrane permeability, efflux-pump overexpression and, most importantly, carbapenemase production [11]. The major carbapenemases detected in *K. pneumoniae* strains worldwide are class A (KPC), class B metallo-β-lactamases (notably NDM) and class D (OXA-48-like) enzymes [9,12,13]. Geographical distribution is highly heterogeneous, with NDM-type carbapenemases dominant in South Asia, KPC in the Americas and parts of Europe, and OXA-48-like enzymes endemic in Turkey, Malta, the Middle East, and North Africa, including Egypt [13,14,15,16]. The burden of CR-KP is increasing in Egypt [17]. Several single-center studies have reported the prevalence of carbapenemase genes among hospitalized patients [17,18,19]. National surveillance data, however, remain fragmented, and multi-center studies directly comparing the distribution of carbapenemase genotypes, antimicrobial susceptibility profiles and, critically, the functional transferability of the underlying resistance plasmids across Egyptian tertiary care settings are still scarce.

In this context, it is of clinical importance to determine whether co-carriage of *bla*_OXA-48_/*bla*_NDM-1_ represents a mobile, conjugatively transferable reservoir rather than clonal expansion alone, with direct implications for infection-control practice and antimicrobial stewardship policy. Therefore, the objectives of this study were to (i) determine the prevalence of carbapenem resistance and MDR/XDR phenotypes among clinical *K. pneumoniae* isolates recovered from two Egyptian tertiary care hospitals over two years, (ii) characterize the major carbapenemase-encoding genes by phenotypic and molecular methods, and (iii) assess the horizontal mobility of carbapenemase-encoding plasmids by conjugation assay, to clarify the drivers of the current CR-KP epidemiology in Egyptian healthcare settings.

## 2. Materials and Methods

### 2.1. Isolation and Identification of K. pneumoniae Isolates

A total of 94 non-duplicate clinical *K. pneumoniae* isolates were included in this study. Isolates were recovered from various clinical specimens submitted to the microbiology units of the central laboratories of two Egyptian tertiary care hospitals across two provinces: El-Demerdash Hospital, Cairo (*n* = 80) and Mabart El-Asafra Hospital, Alexandria (*n* = 14), between September 2021 and October 2023. Isolates were obtained as part of the routine diagnostic microbiology workflow and were not selectively collected on the basis of carbapenem-resistance phenotype; one isolate per patient was retained to avoid duplication. Sampling was markedly unequal between the two sites (El-Demerdash, *n* = 80, 85.1%; Mabart El-Asafra, *n* = 14, 14.9%), reflecting the relative throughput of the two diagnostic laboratories rather than a predefined sampling frame; comparisons between sites are therefore exploratory and are interpreted with caution throughout. Identification of all isolates was confirmed using the automated VITEK^®^ 2 system (bioMérieux, Marcy-l’Étoile, France) and MALDI-TOF mass spectrometry (Bruker Biotyper, Bruker Daltonics, Billerica, MA, USA).

Ethical approval for isolate collection and for the analysis of the accompanying anonymized clinical and demographic data was obtained from the Research Ethics Committee, Faculty of Pharmacy, Cairo University, Egypt (REC-FOPCU; protocol serial number MI (2026), approval date 27 July 2021) prior to specimen collection. Specimens were processed exclusively within the routine diagnostic workflow of the participating hospitals; no additional specimens were collected from patients for research purposes, and no procedure additional to standard clinical care was performed. Because only bacterial isolates were retained and no identifiable patient information was analyzed or reported, the requirement for individual written informed consent was waived by the REC-FOPCU (see Institutional Review Board Statement and Informed Consent Statement).

### 2.2. Antimicrobial Susceptibility Testing and MIC Determination

Antimicrobial susceptibility of all *K. pneumoniae* isolates was determined against 16 antimicrobial agents by the Kirby–Bauer disk diffusion method [20,21] according to the Clinical and Laboratory Standards Institute (CLSI) guidelines [22]. The agents tested were piperacillin (PRL, 100 µg/disk), ampicillin/sulbactam (SAM, 10/10 µg/disk), aztreonam (ATM, 30 µg/disk), amikacin (AK, 30 µg/disk), ceftriaxone (CRO, 30 µg/disk), ceftazidime (CAZ, 30 µg/disk), cefotaxime (CTX, 30 µg/disk), gentamicin (CN, 10 µg/disk), ciprofloxacin (CIP, 5 µg/disk), levofloxacin (LEV, 5 µg/disk), gatifloxacin (GAT, 5 µg/disk), nitrofurantoin (F, 300 µg/disk), imipenem (IPM, 10 µg/disk), meropenem (MEM, 10 µg/disk), colistin (CT, 10 µg/disk) and tigecycline (TGC, 15 µg/disk). Minimum inhibitory concentrations (MICs) were determined using the automated VITEK^®^ 2 system (bioMérieux, France) with AST-GN cards, which included 15 antibiotics: ticarcillin, ticarcillin/clavulanic acid, piperacillin, piperacillin/tazobactam, ceftazidime, cefepime, aztreonam, imipenem, meropenem, amikacin, gentamicin, tobramycin, ciprofloxacin, trimethoprim/sulfamethoxazole and colistin.

The two panels were deliberately not identical. The VITEK^®^ 2 AST-GN card carries a fixed, manufacturer-configured set of antimicrobial agents that cannot be modified by the end user, whereas the disk-diffusion panel was assembled independently so that all nine antimicrobial categories required for the MDR/XDR/PDR classification scheme of Magiorakos et al. [6] were represented, including glycylcyclines (tigecycline) and nitrofurans, which are not available on the AST-GN card. Conversely, agents such as tobramycin and trimethoprim/sulfamethoxazole are supplied on the card but were not included in the disk panel. Importantly, imipenem, meropenem and colistin, the three agents on which the principal conclusions of this study depend, were tested by both methods and additionally confirmed by broth microdilution, so that the difference in panel composition does not affect the carbapenem- and colistin-resistance results reported here. The MICs of imipenem, meropenem and colistin were confirmed by the broth microdilution method. *Escherichia coli* ATCC^®^ 25922 was used as a quality control strain, and results were interpreted according to CLSI breakpoints for Enterobacteriaceae [22], except for colistin and tigecycline, which were interpreted according to EUCAST clinical breakpoints [23]. Isolates were classified as multidrug-resistant (MDR), extensively drug-resistant (XDR) or pandrug-resistant (PDR) according to the interim standard definitions of Magiorakos et al. [6] based on the following antimicrobial categories: penicillins, penicillin/β-lactamase inhibitor combinations, extended-spectrum cephalosporins, monobactams, carbapenems, fluoroquinolones, aminoglycosides, glycylcyclines and polymyxins.

### 2.3. Phenotypic Detection of Carbapenemase Production

Carbapenemase production among CR-KP isolates was assessed using three phenotypic assays, as previously described [9,22,24,25]: the modified Hodge test (MHT) [26], the modified carbapenem inactivation method (mCIM) [22], and the modified Carba NP test (mCNP), performed according to CLSI [22] with a minor modification substituting cetyltrimethylammonium bromide (CTAB) (Sigma-Aldrich, Gillingham, Dorset, UK) for the standard bacterial protein-extraction reagent, as described by Bakour et al. [27].

### 2.4. Molecular Detection and Identification of Carbapenemase-Encoding Genes

All isolates were screened by conventional PCR for the five principal carbapenemase genes (*bla*_KPC_, *bla*_NDM_, *bla*_IMP_, *bla*_VIM_, and *bla*_OXA-48_). Genomic DNA was extracted by the boiling method [28]. Gene-specific primers (Willowfort, Birmingham, UK; Table 1) were used in 25 μL reactions (12.5 μL 2× COSMO PCR RED Master Mix [Willowfort, UK], 1 μL each forward/reverse primer [10 pmol/L], 4 μL DNA template, nuclease-free water to volume), amplified in an Arktik™ Thermal Cycler (Thermo Scientific, Waltham, MA, USA) per the protocol of Poirel et al. [29]. In each PCR run, the following reference strains were used as positive controls: *K. pneumoniae* ATCC^®^ BAA-2146 for *bla*_NDM_, *K. pneumoniae* NCTC 13442 for *bla*_OXA-48_, *K. pneumoniae* ATCC^®^ BAA-1705 for *bla*_KPC_, *K. pneumoniae* NCTC 13440 for *bla*_VIM_, and *E. coli* NCTC 13476 for *bla*_IMP_; nuclease-free water served as the no-template negative control. Amplicons were resolved by electrophoresis (Thermo Scientific, USA) on 2% (*w*/*v*) 1× TAE-agarose gels and visualized under a Syngene™ UV transilluminator (Fisher Scientific, Loughborough, UK) [29]. Positive PCR products were purified using the ExoSAP-IT™ Express PCR product clean-up reagent (Fisher Scientific, UK) prior to Sanger sequencing of a representative subset of purified amplicons on a SeqStudio™ Genetic Analyzer (Thermo Fisher Scientific, Waltham, MA, USA). Sequences were compared against the NCBI non-redundant nucleotide database using BlastN to confirm carbapenemase gene identity and variant. Isolates not selected for sequencing were classified by PCR amplicon size alone; this limitation is addressed in the Discussion (Section 4).

### 2.5. Conjugation Assay for Carbapenemase-Producing K. pneumoniae

The horizontal mobility and transferability of plasmids encoding carbapenemase determinants were assessed by solid-surface (spot) mating conjugation assays, using a representative subset of 18 CRKP clinical isolates as donors and sodium-azide-resistant *E. coli* J53 (J53 Az^R^) as the recipient strain. This subset was selected from the full collection of 94 molecularly characterized isolates so as to span every carbapenemase genotype identified in the collection. The dominant *bla*_NDM-1_/*bla*_OXA-48_ co-carriage genotype (67.0%, *n* = 63) contributed the largest number of donors (*n* = 9), while the rarer genotypes were deliberately over-sampled relative to their prevalence, so that each was adequately represented: all four *bla*_KPC_/*bla*_OXA-48_ co-carriage isolates (4.3%, *n* = 4) were included, together with three single *bla*_OXA-48_ donors (23.4%, *n* = 22) and both single *bla*_NDM-1_ isolates (2.1%, *n* = 2). The single isolate carrying *bla*_KPC_ alone (1.1%, *n* = 1) was carbapenem-susceptible and therefore fell outside the carbapenem-resistant donor set. All assays were performed in independent biological triplicate.

Donor and recipient strains were grown overnight in Luria–Bertani (LB) broth at 37 °C, subcultured 1:100 into fresh broth, and grown to mid-log phase (OD600 ≈ 0.6). Donor and recipient suspensions were mixed in a 1:9 volume ratio, harvested, pelleted and resuspended in sterile phosphate-buffered saline (PBS) and spot-inoculated onto non-selective LB agar for 18 h at 37 °C. A nine-fold recipient excess (1:9 donor:recipient by volume) was used in preference to an equal or donor-excess ratio for two reasons. First, because conjugation frequency is expressed per recipient cell (T/R), maintaining recipients at non-limiting density makes transfer donor-limited, so that the resulting ratio reflects the conjugative capacity of the donor strain rather than the availability of recipient cells; under donor excess, the recipient pool becomes limiting, and the T/R ratio saturates, compressing differences between efficient and inefficient donors. Second, a recipient excess approximates the numerical minority of resistant donor organisms relative to susceptible and commensal recipients in the settings in which horizontal transfer is presumed to occur. Because a recipient excess yields lower absolute frequencies than donor-saturated conditions, the conjugation frequencies reported here should be regarded as conservative estimates of transfer capacity. Growth was harvested, resuspended in physiological saline, serially diluted and plated onto selective LB agar containing sodium azide (100 μg/mL, to counter-select donors) and meropenem (1 μg/mL, to select for plasmid acquisition). The meropenem concentration was set at 1 μg/mL because this lies at the CLSI susceptible breakpoint for Enterobacteriaceae and therefore counter-selects the plasmid-free recipient, while remaining low enough not to inhibit transconjugants that have acquired a carbapenemase but retain the comparatively low MICs typical of an *E. coli* J53 background; a higher concentration would risk scoring true transconjugants as negative.

Conjugation frequency was calculated as transconjugant CFU/mL divided by final recipient CFU/mL (T/R ratio). Transfer efficiency was defined as the percentage of donor strains producing ≥ 1 confirmed transconjugant over the total number of donors tested. Donor-alone and recipient-alone suspensions were plated in parallel on the same selective medium as controls for spontaneous resistance; no growth was recovered from either control.

These controls were included in every mating experiment and in every biological replicate, establishing that neither spontaneous meropenem resistance in the recipient nor spontaneous sodium-azide resistance in the donor contributed to the transconjugant counts reported. Putative transconjugants were additionally required to satisfy three independent criteria before acceptance: growth on the dual-selective medium, PCR confirmation of the donor carbapenemase gene content, and carbapenem non-susceptibility by broth microdilution.

Transconjugants were subcultured to purity and confirmed for carbapenemase gene acquisition by multiplex/singleplex PCR (*bla*_KPC_, *bla*_NDM_, *bla*_OXA-48_). Resistance transfer was confirmed phenotypically by broth microdilution susceptibility testing against meropenem, imipenem and ertapenem according to CLSI standards.

### 2.6. Plasmid DNA Extraction

Plasmid DNA from donor CRKP isolates and corresponding transconjugants was extracted using the QIAGEN Plasmid Extraction Kit (QIAGEN, Hilden, Germany) according to the manufacturer’s instructions with minor modifications to optimize the recovery of high-molecular-weight plasmids. Isolates were grown overnight in LB broth at 37 °C; cells were pelleted (8000× *g*, 5 min) and lysed under alkaline conditions. After neutralization and centrifugation to remove chromosomal DNA and debris, the supernatant was added to a silica-based spin column, washed, and eluted in pre-warmed elution buffer. DNA concentration and purity (A260/A280) were determined using a NanoDrop spectrophotometer (Thermo Fisher Scientific, Wilmington, DE, USA). Purified plasmid preparations were stored at −20 °C for subsequent PCR.

### 2.7. Electrophoretic Verification of Plasmid DNA

Plasmid profiles from donor isolates and transconjugants were analyzed by 0.7% agarose gel electrophoresis in 1× Tris-acetate-EDTA (TAE) buffer at 80 V for 90 min, to assess plasmid integrity and to screen for gross chromosomal DNA carry-over (visualized as a high-molecular-weight band or a high-abundance genomic smear retained in or near the loading wells). Gels were stained with a nucleic-acid gel stain and visualized by UV transillumination prior to downstream molecular assays. Agarose electrophoresis of this kind can exclude only gross chromosomal carry-over; it has limited sensitivity for low-level residual chromosomal DNA and was therefore used as a quality-control screen rather than as evidence that preparations were free of chromosomal DNA.

### 2.8. PCR Amplification of Carbapenemase-Encoding Genes in Donors and Transconjugants

Plasmid-borne localization of carbapenemase determinants was assessed by PCR of extracted plasmid DNA from donor isolates and transconjugants, screening for *bla*_OXA-48_, *bla*_NDM_, and *bla*_KPC_. Reactions (20 μL final volume: 10 μL 2× PCR Master Mix [Thermo Fisher Scientific, Waltham, MA, USA], 1 μL each primer [10 pmol/L], 2 μL plasmid DNA template, nuclease-free water to volume) were cycled with initial denaturation at 95 °C for 5 min; 35 cycles of 95 °C/30 s, primer-specific annealing temperature/30 s, 72 °C/45 s; and a final extension at 72 °C for 5 min. Amplicons were resolved by agarose gel electrophoresis. Because alkaline-lysis plasmid preparations may retain trace chromosomal DNA, amplification of a carbapenemase gene from such a preparation is consistent with, but does not by itself establish, plasmid localization; the inference of plasmid carriage in this study therefore rests principally on transfer of the corresponding genes into a plasmid-free recipient by conjugation.

### 2.9. Statistical Analysis

Data were analyzed using Python (version 3.12; pandas and SciPy libraries). Categorical variables (e.g., resistance phenotype, carbapenemase gene carriage, phenotypic test results) were expressed as frequencies and percentages, and continuous variables (e.g., patient age) as mean ± standard deviation. Associations between categorical variables, specifically hospital site, ward type (ICU versus non-ICU), and patient sex versus carbapenem-resistance status, and hospital site versus *bla*_OXA-48_/*bla*_NDM-1_ co-carriage were assessed using the chi-square test or, where expected cell counts were <5, Fisher’s exact test; a two-tailed *p*-value < 0.05 was considered statistically significant. Concordance between phenotypic carbapenemase production (positive by at least one of MHT, mCIM, or mCNP) and genotypic detection (≥1 carbapenemase gene by PCR) among the carbapenem-resistant isolates was expressed as the percentage of isolates with concordant phenotypic and genotypic results. The 95% confidence interval for the plasmid conjugation transfer efficiency was calculated using the exact (Clopper–Pearson) binomial method, and the 95% confidence interval for the odds ratio describing hospital-site variation in *bla*_OXA-48_/*bla*_NDM-1_ co-carriage was calculated using the Woolf logit method. As this was an observational surveillance study encompassing all isolates meeting inclusion criteria during the study period, no a priori sample-size or power calculation was performed; this is acknowledged as a limitation in the Discussion (Section 4).

Given the size of the collection and the marked imbalance between the two participating sites, all association tests should be regarded as exploratory and hypothesis-generating rather than confirmatory; they are reported without adjustment for multiple comparisons, and the absence of a statistically significant association should not be read as evidence of no association.

## 3. Results

### 3.1. Clinical Characteristics of K. pneumoniae Isolates

**Figure 1:**
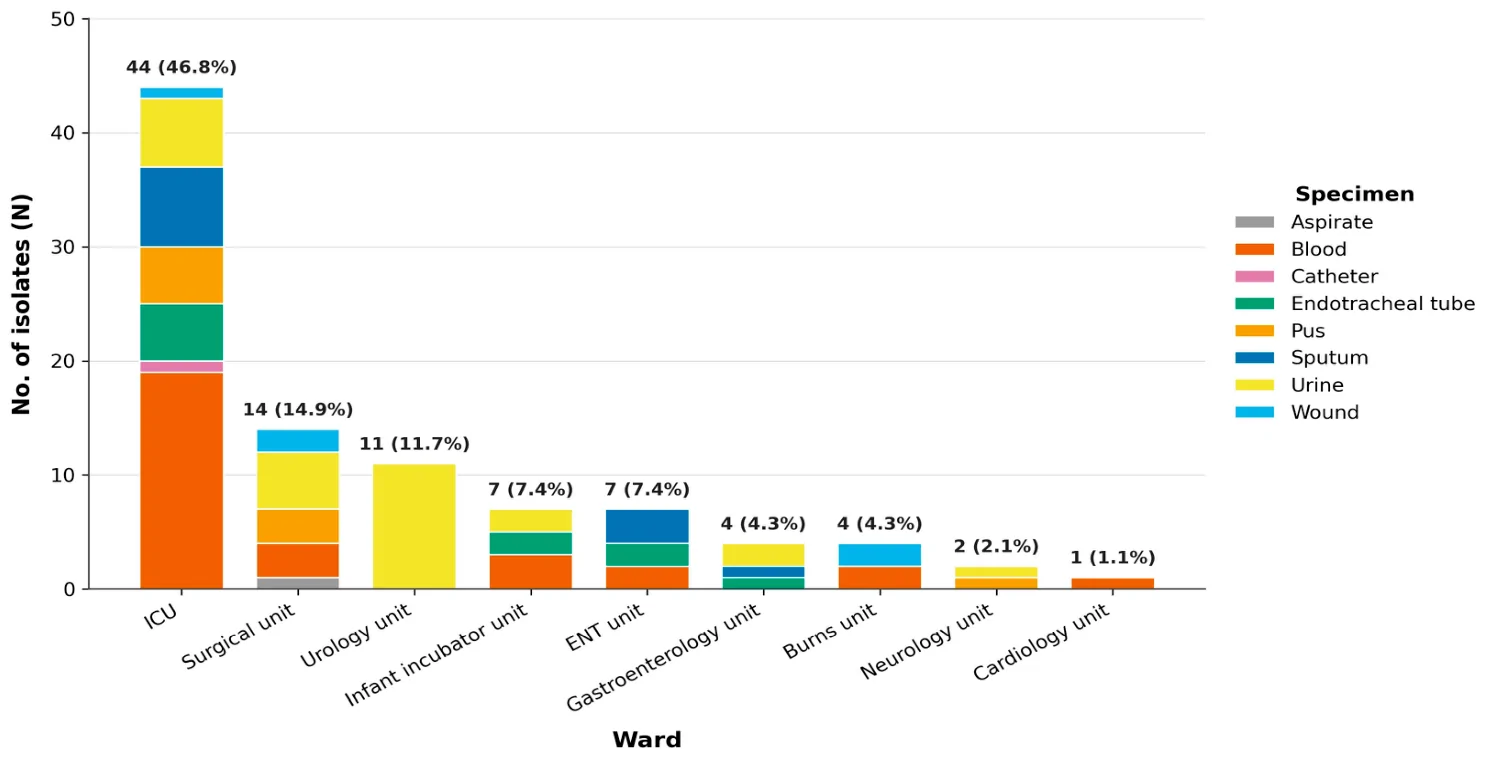
Distribution of clinical *K. pneumoniae* isolates (*N* = 94) by hospital ward and specimen type. Each bar represents one ward; the value above each bar is the total number of isolates recovered from that ward and its percentage of the full collection. Bars are subdivided by specimen source according to the color key. Created with Python version 3.12.3 (matplotlib 3.10.8).

Overall, 94 isolates of *K. pneumoniae* were isolated from different clinical departments, with the intensive care units (ICUs) being the most common source (46.8%), followed by the surgical (14.9%), urology (11.7%), infant incubator (7.4%) and ear, nose, and throat (ENT) (7.4%) units (Figure 1). The most frequent sources of specimens were blood (31.9%) and urine (28.7%), followed by sputum (11.7%) and endotracheal aspirates (10.6%), which together accounted for 83.0% of all isolates; the remaining 17.0% were from other clinical sources. Detailed demographic and clinical data of individual isolates are shown in Supplementary Table S1, and summary statistics of sex and age distribution in Table 2. All 94 clinical isolates were identified as *K. pneumoniae* subsp. *pneumoniae* by the automated VITEK^®^ 2 system (99% confidence) and confirmed by MALDI-TOF MS (Bruker Biotyper; all scores ≥ 2.0) with complete concordance between both identification methods.

### 3.2. Antibiogram Profiles of Tested K. pneumoniae Isolates

**Figure 2:**
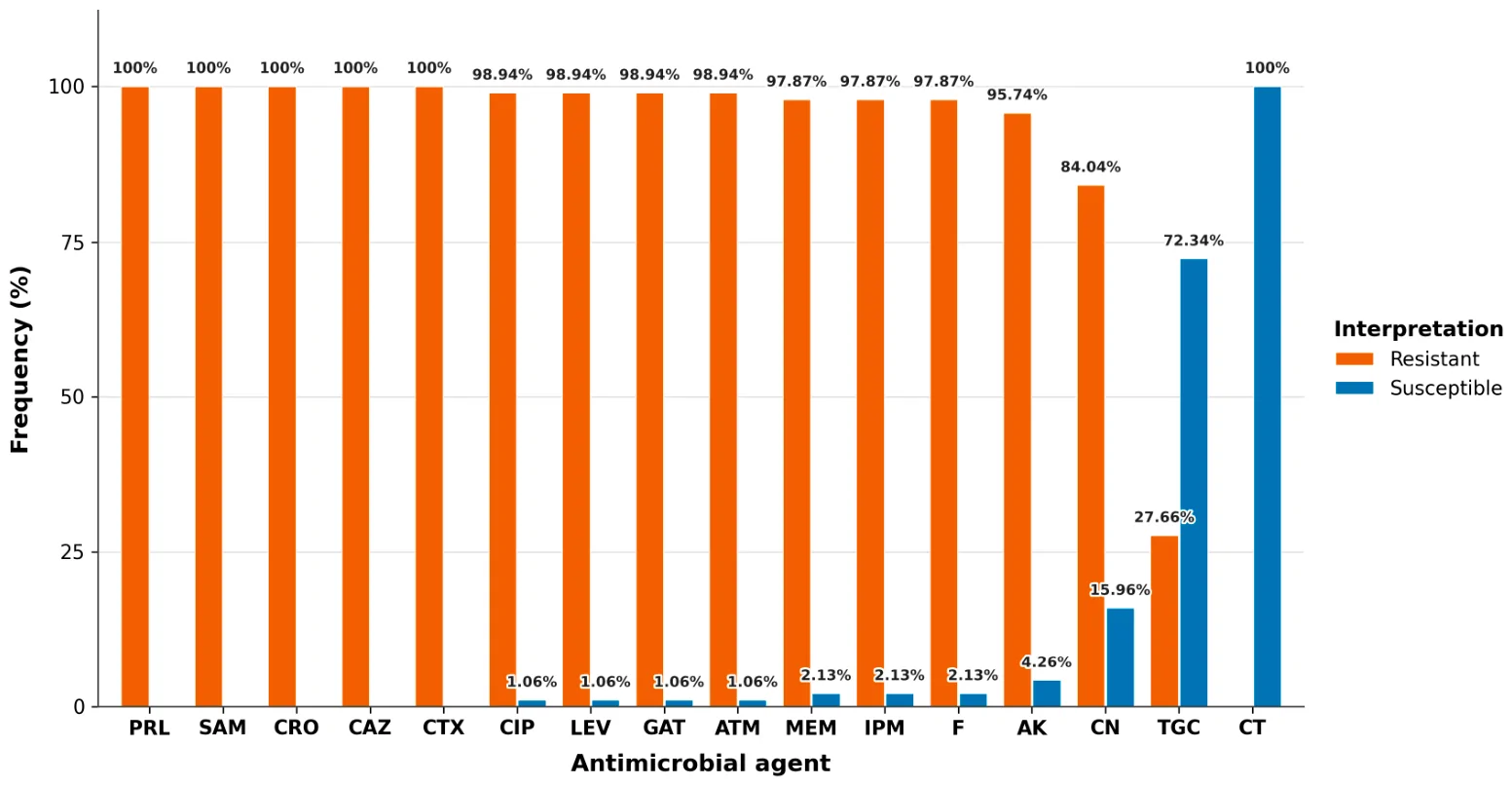
Antibiogram of tested *K. pneumoniae* isolates (*N* = 94) against 16 antibiotics by the Kirby–Bauer disk diffusion method (PRL, piperacillin; SAM, ampicillin/sulbactam; CRO, ceftriaxone; CAZ, ceftazidime; CTX, cefotaxime; CIP, ciprofloxacin; LEV, levofloxacin; GAT, gatifloxacin; ATM, aztreonam; MEM, meropenem; IPM, imipenem; F, nitrofurantoin; AK, amikacin; CN, gentamicin; TGC, tigecycline; CT, colistin). For each agent, paired bars show the percentage of isolates interpreted as resistant and as susceptible; no isolate was interpreted as intermediate by disk diffusion, so the two categories sum to 100% for every agent. Results were interpreted per CLSI breakpoints (M100-S28, 2018), except for colistin and tigecycline, interpreted per EUCAST breakpoints (version 8.1, 2018). Created with Python version 3.12.3 (matplotlib 3.10.8).

As shown in Figure 2, the isolates exhibited high resistance across a wide range of antimicrobial agents. Almost all the isolates exhibited uniform resistance to β-lactams (piperacillin, ampicillin/sulbactam, ceftriaxone, ceftazidime, cefotaxime, aztreonam; 98.94–100%) and carbapenems (imipenem/meropenem; 97.87%). Resistance to fluoroquinolones was observed in 98.94% of isolates and to nitrofurantoin in 97.87%. High resistance was also observed to aminoglycosides (amikacin 95.74%, gentamicin 84.04%), while resistance to tigecycline was moderate (27.66%). Colistin results obtained by disk diffusion are shown in Figure 2 for completeness only; disk diffusion is not a valid method for colistin because of the poor diffusion of polymyxins in agar, and colistin susceptibility in this study was determined by broth microdilution, which is the reference method and the basis for all colistin conclusions reported below.

### 3.3. MIC Results of Tested K. pneumoniae Isolates

**Figure 3:**
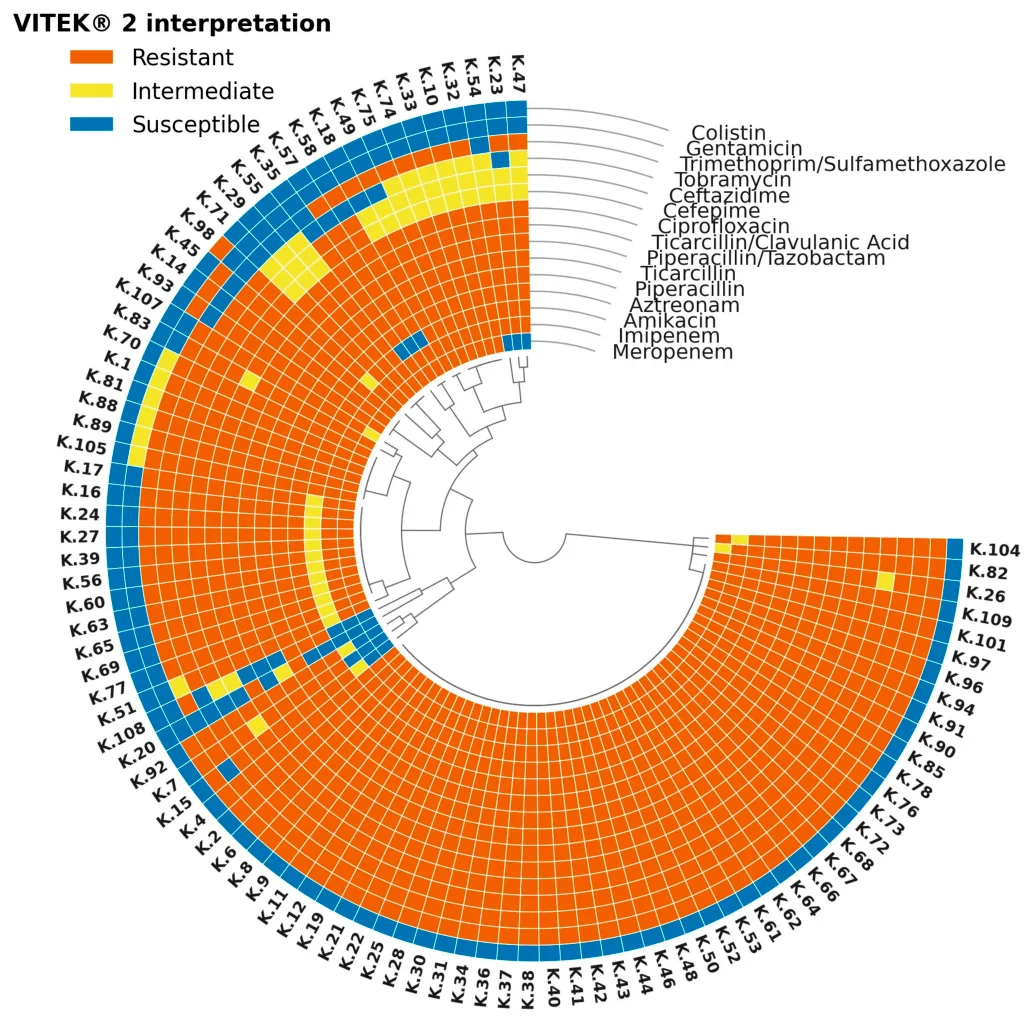
Circular heatmap of antimicrobial susceptibility profiles for all 94 clinical *K. pneumoniae* isolates based on VITEK^®^ 2 AST against the 15 agents supplied on the AST-GN card. Each concentric ring represents one antimicrobial agent and each radial sector one isolate; vermillion indicates resistant, yellow intermediate, and blue susceptible. Isolates and antimicrobials were ordered by hierarchical clustering (Ward linkage on Euclidean distances); the inner dendrogram shows the clustering of isolates. Created with Python version 3.12.3 (matplotlib 3.10.8, SciPy 1.17.1).

VITEK^®^ 2 MIC results demonstrated a substantial resistance burden for most of the agents evaluated (Figure 3), with β-lactams demonstrating the highest resistance followed by quinolones and aminoglycosides. Eighty-nine isolates (94.68%) were non-susceptible to imipenem (88 resistant, 1 intermediate), and 86 isolates (91.49%) were non-susceptible to meropenem (84 resistant, 2 intermediate). Only one isolate (1.06%) was identified as colistin-resistant by VITEK^®^ 2; broth microdilution confirmed the imipenem/meropenem results and did not identify colistin resistance among the isolates tested. Because broth microdilution is the reference method for polymyxins, the collection is reported here as uniformly colistin-susceptible on that basis, and the single VITEK^®^ 2 colistin-resistant result (isolate K.98) is regarded as unconfirmed.

Hierarchical clustering of the susceptibility profiles (Figure 3) separated the antimicrobial agents into four groups that reflect the degree of retained activity. Colistin formed a single outlier branch, with only one resistant isolate (1.1%), and gentamicin formed a second isolated branch (58.5% resistant), making these the two agents with the greatest retained activity across the collection. Ceftazidime, cefepime, tobramycin, and trimethoprim/sulfamethoxazole grouped together at intermediate resistance levels (80.9–87.2% resistant), while the remaining nine agents, including imipenem, meropenem, and amikacin, formed a single tight cluster of uniformly high resistance (77.7–100% resistant). At the isolate level, clustering divided the collection into two clades: a larger clade of 49 isolates that were resistant to every agent tested except colistin, and a smaller clade of 45 isolates retaining variable activity, most notably to gentamicin (73.3% susceptible) and, to a lesser extent, to meropenem (17.8% susceptible) and trimethoprim/sulfamethoxazole (24.4% susceptible).

### 3.4. Antimicrobial Resistance Patterns: MDR and XDR Classification

All tested isolates (100%) were multidrug-resistant (MDR) as defined by non-susceptibility to at least one agent in three or more antimicrobial categories according to AST using the automated VITEK^®^ 2 system and the acquired-resistance criteria of Magiorakos et al. [6]. In total, 94.68% (89/94) of isolates were non-susceptible to imipenem and/or meropenem. According to the criteria of Magiorakos et al. [6] for all nine antimicrobial categories evaluated in this study, the isolates were divided into two groups: isolates in Group I (*n* = 89, 94.7%) were non-susceptible to at least one agent in every antimicrobial category tested except for polymyxins (100% colistin-susceptible) and glycylcyclines (72.34% tigecycline-susceptible) meeting the interim standard definition of extensively drug-resistant (XDR) rather than MDR alone; and Group II (5 isolates, 5.3%) remained MDR and carbapenem-susceptible. This reclassification at the category level more accurately reflects the degree of cross-class resistance observed in Figure 2 and Figure 3 than would a blanket MDR designation.

### 3.5. Phenotypic Identification of Carbapenem-Resistance Mechanisms

**Figure 4:**
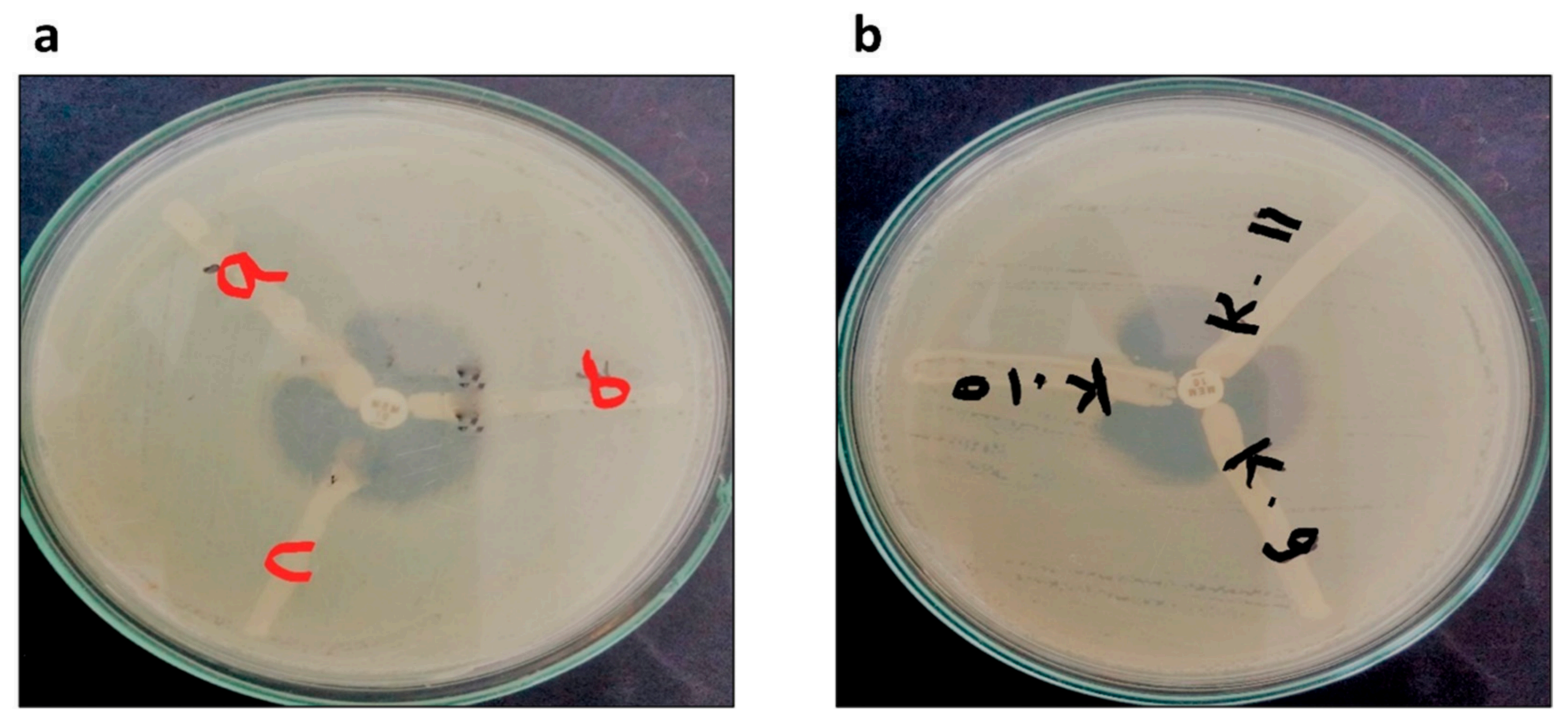
Modified Hodge test (MHT) results on Mueller–Hinton agar. (**a**) Screening plate showing three test streaks, labeled a, b and c, drawn radially outward from a central meropenem (10 µg) disk on a lawn of *E. coli* ATCC^®^ 25922: streak a, tested *K. pneumoniae* isolate (MHT positive); streak b, MHT positive control (*K. pneumoniae* ATCC^®^ BAA-1705™); streak c, MHT negative control (*K. pneumoniae* ATCC^®^ BAA-1706™). A positive result is read as a clover-leaf-shaped distortion of the inhibition zone where the test streak meets the *E. coli* lawn, produced by local hydrolysis of meropenem; the negative control produces no such distortion. (**b**) Representative plate for clinical isolates K.9, K.10 and K.11, all three of which show the characteristic clover-leaf indentation and were therefore recorded as MHT positive.

**Figure 5:**
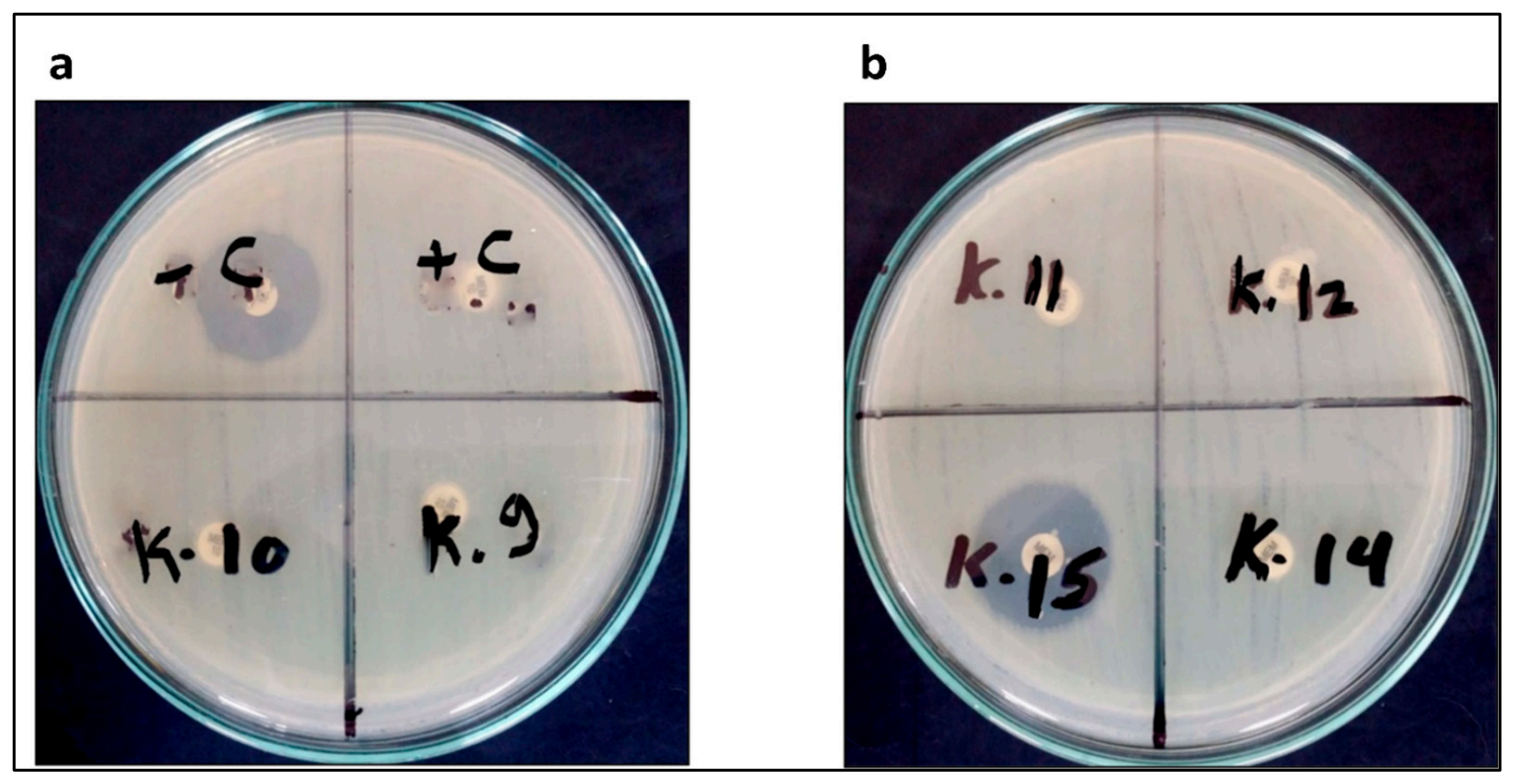
Modified carbapenem inactivation method (mCIM) results on Mueller–Hinton agar, read as the inhibition-zone diameter around a meropenem (10 µg) disk previously incubated with the test organism. Carbapenemase-producing isolates hydrolyze the meropenem, so no inhibition zone develops (positive result), whereas non-producers leave the disk active, and a zone of >19 mm is retained (negative result). (**a**) Negative control (−C; *K. pneumoniae* ATCC^®^ BAA-1706, upper left), positive control (+C; *K. pneumoniae* ATCC^®^ BAA-1705, upper right) and clinical isolates K.10 (lower left) and K.9 (lower right); the negative control shows a clearly retained inhibition zone, while the positive control and both clinical isolates show none and were recorded as mCIM positive. (**b**) Clinical isolates K.11 (upper left), K.12 (upper right), K.15 (lower left) and K.14 (lower right); K.11, K.12 and K.14 show no inhibition zone and were recorded as mCIM positive, whereas K.15 retains a zone and was recorded as mCIM negative, consistent with its carbapenem-susceptible phenotype (Supplementary Table S2).

**Figure 6:**
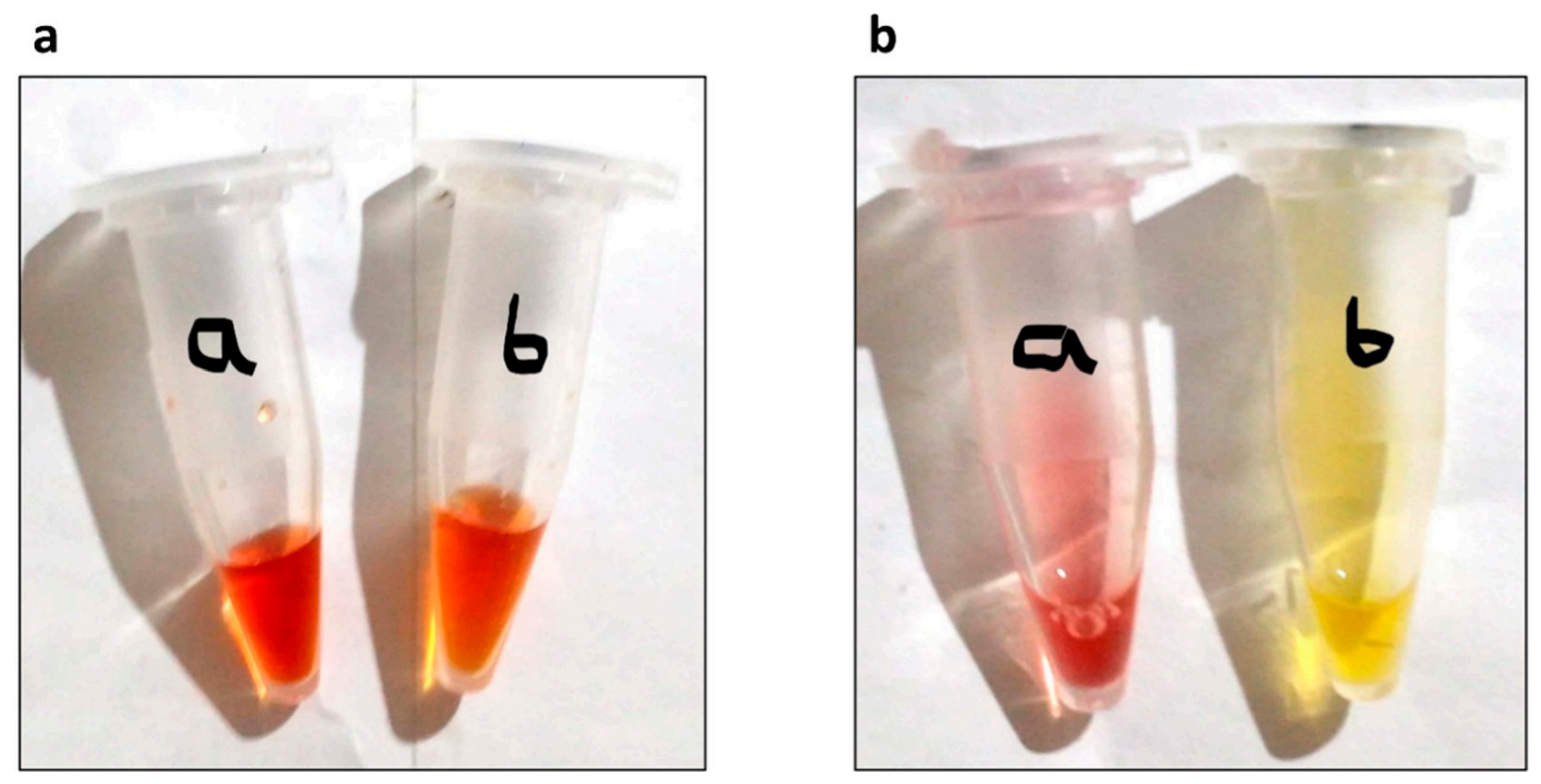
Modified Carba NP test results, interpreted as a color change of the phenol red pH indicator. Each isolate is screened in two tubes. Tube a contains the *K. pneumoniae* protein extract with phenol red indicator solution but without a carbapenem substrate and serves as the internal color control. Tube b contains the same extract plus imipenem. The hydrolysis of imipenem by a carbapenemase produces protons, which lowers the pH and changes the indicator in tube b from orange-red to yellow. (**a**) Negative result: tubes a and b remain orange-red, indicating no carbapenemase activity. (**b**) Positive result: tube a remains red while tube b has turned yellow, indicating carbapenemase activity. Panel letters (**a**,**b**) refer to the two results shown. The lower-case letters a and b marked on the individual tubes refer to the substrate-free and imipenem-containing reactions within each result.

Phenotypic assays identified 88 of the 89 CR-KP isolates (98.9%) as definitive carbapenemase producers (positive by at least one of MHT, mCIM, or mCNP); one isolate (K.104) yielded only an intermediate mCIM result with no other positive phenotypic test and was therefore not classified as a definitive producer. Individually, 68 isolates (76.4%) were MHT-positive (Figure 4), 87 isolates (97.8%) were mCIM-positive (excluding two isolates, K.1 and K.104, with an intermediate mCIM result; Figure 5), and 84 isolates (94.4%) were mCNP-positive (Figure 6).

### 3.6. Genotypic Distribution of Carbapenemase-Encoding Genes

**Figure 7:**
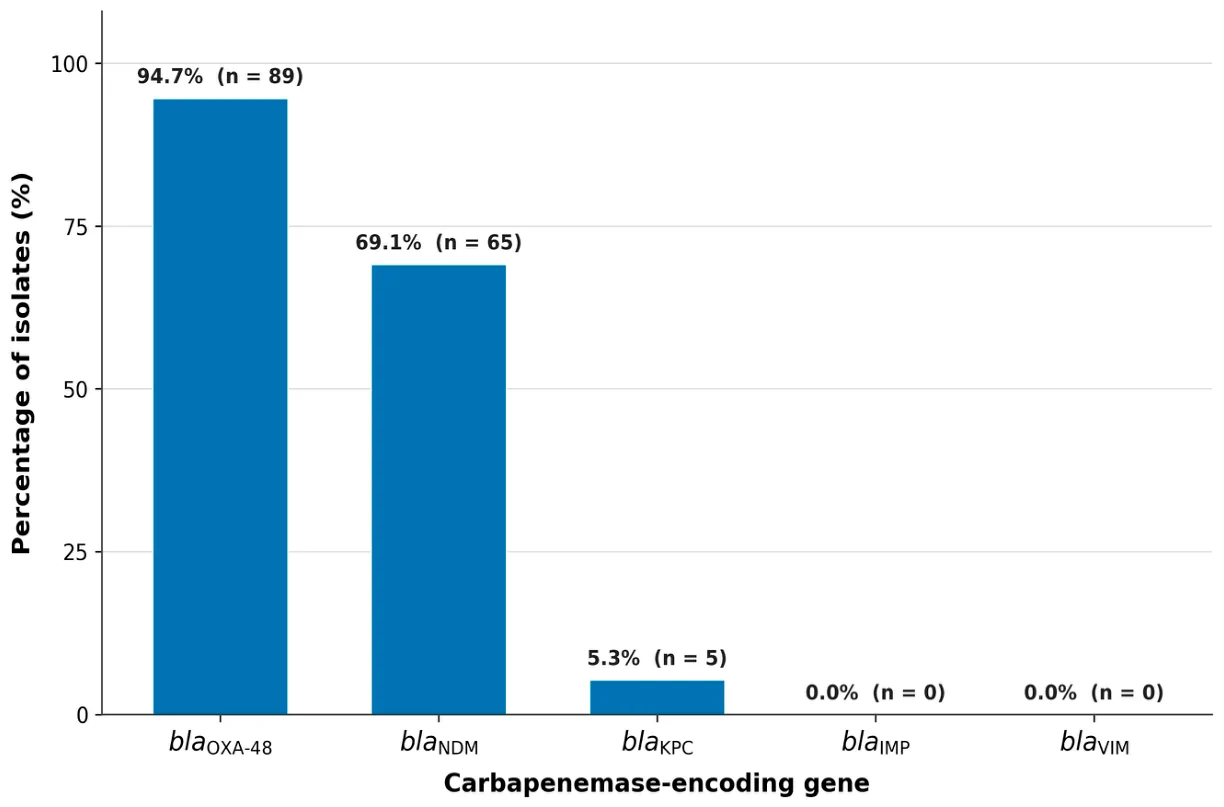
Prevalence of carbapenemase genes among the tested *K. pneumoniae* isolates (*N* = 94) based on PCR results. Bars show the percentage of isolates in which each gene was detected, alone or in combination with another carbapenemase gene, with the corresponding number of isolates given in parentheses. All five genes included in the PCR panel are shown; *bla*_IMP_ and *bla*_VIM_ were not detected in any isolate. Created with Python version 3.12.3 (matplotlib 3.10.8).

PCR screening for carbapenemase-encoding genes (Supplementary Figures S1 and S2) identified single or mixed carbapenemase genes in 92 of 94 isolates (97.9%; Table 3). *bla*_OXA-48_ was the most frequently detected gene, followed by *bla*_NDM_ and *bla*_KPC_ (Figure 7); *bla*_IMP_ and *bla*_VIM_ were not identified in any isolate.

### 3.7. DNA Sequencing Analysis

Sanger sequencing and BlastN analysis of representative purified PCR-positive amplicons confirmed the PCR results and identified *bla*_OXA-48_ and *bla*_NDM-1_ as the carbapenemase gene variants present; isolates harboring *bla*_KPC_ were identified by PCR amplicon size alone and were not selected for sequencing. The *bla*_OXA-48_ and *bla*_NDM-1_ sequences were deposited in GenBank under accession numbers PZ251032–PZ251036 and PZ482034–PZ482035, respectively. The full antimicrobial resistance, phenotypic, and genotypic profiles of all isolates are summarized in Supplementary Table S2.

### 3.8. Gel Electrophoretic Verification of Plasmid DNA

Plasmid extraction from the donor strains and their transconjugants was confirmed by 0.7% agarose gel analysis. Agarose gel analysis showed distinct high-molecular-weight bands corresponding to intact conjugative plasmids, with no chromosomal DNA smear in or near the loading wells. Gross chromosomal carry-over was therefore excluded, and the preparations were suitable for downstream PCR, though a gel of this kind cannot rule out low-level residual chromosomal DNA (Section 2.7).

### 3.9. Genetic Context and Transferability of Carbapenemase-Encoding Plasmids

Successful horizontal transfer of carbapenemase-harboring plasmids from the representative CRKP donor subset (*n* = 18) to *E. coli* J53 (J53 Az^R^) was demonstrated by conjugation assays performed in independent biological triplicate. Plasmids were transferred successfully from 77.8% (14/18) of donor isolates, with an overall transfer efficiency of 77.8% (exact 95% CI: 52.4–93.6%, Clopper–Pearson method). For positive donors, the mean conjugation frequency was (3.42 ± 0.81) × 10^−3^ transconjugants per recipient cell (individual strains ranged from 2.1 × 10^−5^ to 6.5 × 10^−3^) and was highly reproducible across independent replicates. PCR confirmed that each transconjugant acquired the carbapenemase gene content of its corresponding donor: *bla*_NDM-1_ and *bla*_OXA-48_ were co-transferred from donors harboring both determinants, *bla*_KPC_ and *bla*_OXA-48_ were likewise co-transferred from the *bla*_KPC_/*bla*_OXA-48_ co-carriage donors, and donors carrying a single determinant transferred that gene alone (*bla*_OXA-48_ or *bla*_NDM-1_). All validated transconjugants were non-susceptible to meropenem, imipenem and ertapenem (MIC phenotypes), consistent with their parental CRKP donor strains, confirming that the mobilized plasmids confer clinically relevant carbapenem resistance along with associated co-resistance determinants.

Transfer outcomes are summarized by donor genotype in Table 4. Transconjugants were recovered from eight of the nine *bla*_OXA-48_/*bla*_NDM-1_ co-carriage donors (88.9%), from three of the four *bla*_KPC_/*bla*_OXA-48_ co-carriage donors (75.0%), from two of three single *bla*_OXA-48_ donors and from 1 of 2 single *bla*_NDM-1_ donors. Per-donor transfer efficiency was therefore numerically higher among co-carriage donors than among donors carrying a single determinant (11/13 versus 3/5), although this difference was not statistically significant (Fisher’s exact *p* = 0.533), and the small number of single-determinant donors precludes any firm comparison between genotype groups.

### 3.10. Plasmid Association and Distribution of Carbapenemase-Encoding Genes

**Figure 8:**
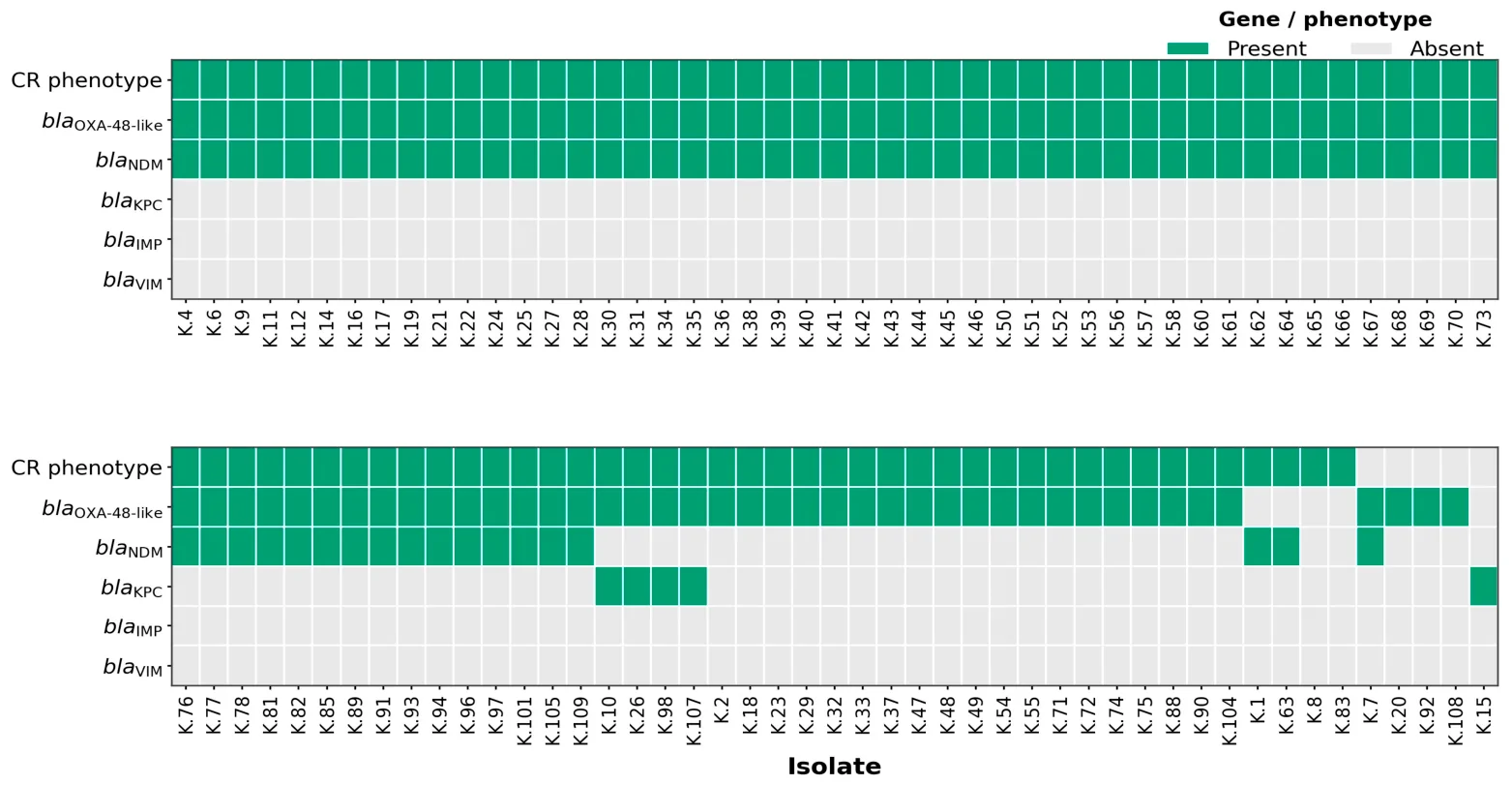
Heatmap of genotype–phenotype correlation of carbapenem resistance in all 94 clinical *K. pneumoniae* isolates. Each column represents one isolate and each row one carbapenemase-encoding gene (*bla*_OXA-48-like_, *bla*_NDM_, *bla*_KPC_, *bla*_IMP_, *bla*_VIM_) or the phenotypic carbapenem-resistance status (CR phenotype). Green indicates presence of the gene or phenotype, and gray indicates absence. The 94 isolates are divided across the stacked panels for legibility and are ordered by genotype pattern, so that strains sharing the same carbapenemase-gene combination appear together. Created with Python version 3.12.3 (matplotlib 3.10.8).

Conjugation data, supported by PCR of plasmid preparations, indicate that at least a proportion of the carbapenemase genes in this collection are carried on conjugative plasmids. The strongest evidence is functional rather than physical: transfer of the corresponding genes into a plasmid-free *E. coli* recipient. Direct physical localization by long-read sequencing, plasmid sequencing or replicon typing was not performed, and the plasmid carriage of genes in donors that did not transfer remains formally undetermined. Of the 94 isolates tested, *bla*_OXA-48_ was the most frequent carbapenemase gene, detected alone or in combination in the large majority of strains; dual co-carriage of *bla*_NDM-1_ and *bla*_OXA-48_ was the dominant resistance genotype, present in 67.0% (63/94) of isolates. Single-determinant *bla*_OXA-48_ carriage occurred in 23.4% (22/94) of isolates; co-carriage of *bla*_KPC_ and *bla*_OXA-48_ occurred in 4.3% (4/94); and single *bla*_NDM-1_ (2.1%, 2/94) and single *bla*_KPC_ (1.1%, 1/94) carriage were less common (Figure 8). The high frequency of *bla*_NDM-1_/*bla*_OXA-48_ co-occurrence, combined with the conjugative transferability demonstrated in the donor subset, is consistent with the presence of a mobile reservoir of carbapenemase-encoding plasmids in these settings. Because the conjugation assays were restricted to 18 donors and no plasmid typing was undertaken, the extent of that reservoir across the full collection cannot be quantified from these data.

### 3.11. Statistical Associations

To formally test whether carbapenem-resistance prevalence and carbapenemase-genotype distribution differed by hospital site, ward type, or patient sex, chi-square/Fisher’s exact tests were applied to the isolate-level dataset (*n* = 94; Supplementary Tables S1 and S2). Carbapenem resistance was numerically more frequent at El-Demerdash Hospital (77/80, 96.3%) than at Mabart El-Asafra Hospital (12/14, 85.7%), but this difference was not statistically significant (Fisher’s exact *p* = 0.159). Similarly, carbapenem resistance was not significantly associated with ICU admission (43/44, 97.7%, in ICU isolates versus 46/50, 92.0%, in non-ICU isolates; Fisher’s exact *p* = 0.367) or with patient sex (38/41, 92.7%, in females versus 51/53, 96.2%, in males; Fisher’s exact *p* = 0.650). These findings indicate that the very high carbapenem-resistance prevalence observed in this collection was broadly distributed across hospital sites, ward types, and patient demographics, rather than concentrated within a specific subgroup, consistent with an endemic, rather than a localized outbreak-associated, resistance pattern.

Co-carriage of *bla*_OXA-48_ and *bla*_NDM-1_ likewise did not differ significantly by hospital site (55/80, 68.8%, at El-Demerdash Hospital versus 8/14, 57.1%, at Mabart El-Asafra Hospital; Fisher’s exact *p* = 0.539; odds ratio [El-Demerdash relative to Mabart El-Asafra] = 1.65, 95% CI: 0.51–5.31), indicating that this dominant dual-carriage genotype is similarly distributed across both participating tertiary care centers, rather than reflecting a single-site clonal cluster.

Concordance between phenotypic carbapenemase production and PCR-based genotypic detection among the 89 carbapenem-resistant isolates was high, with 86/89 isolates (96.6%) showing concordant phenotypic and genotypic results (Table 5). The three discordant isolates comprised two that were phenotypically positive but negative for all five genes screened (K.8 and K.83) and one that was genotypically positive but yielded only an intermediate mCIM result with no other positive phenotypic test (K.104). These are discussed in Section 4 in the context of established mechanisms of phenotypic–genotypic discordance in carbapenemase testing. It should be noted that this analysis is restricted to the 89 carbapenem-resistant isolates, since the phenotypic carbapenemase assays were performed only on that subgroup (Section 2.3); the five carbapenem-susceptible isolates, all of which carried at least one carbapenemase gene, are considered separately in Section 4.

## 4. Discussion

Antimicrobial resistance (AMR) is a serious public health threat, especially in developing countries [30,31]. *K. pneumoniae* is associated with high morbidity and mortality in hospitalized patients, especially in ICU settings, and shows multidrug resistance worldwide [32,33]. For years, carbapenems have been effective against MDR *K. pneumoniae* [34], but CR-KP strains have become prevalent worldwide in recent years [9], and the burden remains high in Mediterranean countries, especially Egypt [13,35,36]. Thus, the present study aimed to investigate the prevalence of carbapenem resistance, the main carbapenemase genes and the horizontal transferability of the underlying resistance plasmids in clinical *K. pneumoniae* isolates from two tertiary care Egyptian hospitals. The majority of isolates were recovered from the ICUs (46.8%). This is in line with the reports of Li et al. [37] and Hafiz et al. [38], who reported recovery rates of 41.5% and 42% from the ICUs, respectively. Blood (31.9%) was the predominant specimen source, followed by urine (28.7%) and sputum (11.7%), which was similar to that reported by Romanus et al. [39] (blood, 40%; urine, 30.5%; sputum, 23.6%) and probably reflects the role of *K. pneumoniae* in bloodstream infections among hospitalized or immunocompromised patients [40]. By comparison, Wang et al. [41] reported sputum as the main source (74%). Males were predominant (56.4%) in this study, which is consistent with Osama et al. [42] in Egypt and Alhazmi et al. [43] in Saudi Arabia, and most patients (77.7%) were adults over 15 years of age, similar to Hafiz et al. [38]. The antibiogram showed 100% resistance to the β-lactams (piperacillin, ampicillin/sulbactam, ceftriaxone, ceftazidime, and cefotaxime) and 98.94% to aztreonam, which is slightly higher than that reported by Al-Baz et al. [44] in Egypt and Oliveira et al. [45] in Portugal. The carbapenem resistance (97.87% to imipenem and meropenem by disk diffusion) was consistent with Hassuna et al. [46] (>95%) and Abdelaziz [47] (93.4%) in Egypt. High quinolone (98.94%) and nitrofurantoin (97.87%) resistance was concordant with Afify et al. [48], but quinolone susceptibility was higher in other Egyptian studies [42,44,46]. The aminoglycoside resistance (95.74% for amikacin, 84.04% for gentamicin) was comparable to the resistance described in Egypt (79.55%, 71.74%) [48] and Saudi Arabia (100%, 98%) [49], but lower than the other Egyptian studies [42,44].

Tigecycline resistance was limited (27.66%), comparable to South Korea (37.8%) [50] and Egypt (39.1%) [51]. All isolates remained colistin-susceptible, consistent with Ghaith et al. [17], although recent Egyptian and international studies report a rising prevalence of colistin resistance [48,52,53,54,55]. Colistin (100% susceptibility) and tigecycline (72.34% susceptibility) therefore remain the most active agents against CR-KP in this setting, positioning them as last-resort options, in agreement with prior work [42,55].

Using the VITEK^®^ 2 system and the acquired-resistance criteria of Magiorakos et al. [6], all isolates (100%) met the MDR definition, consistent with Tawfick et al. [18] (98.7%) and Ibrahim et al. [56] (95.2%) in Egypt. Applying the same framework at the category level, however, the carbapenem-resistant subgroup (Group I, *n* = 89, 94.7%) remained susceptible only to polymyxins and glycylcyclines and was therefore non-susceptible to at least one agent in every other tested category, meeting the interim standard definition of extensively drug-resistant (XDR) rather than MDR alone. This refined classification aligns this cohort more closely with the 83% XDR rate reported by Hassuna et al. [46] in Egypt than a blanket MDR estimate would suggest and reinforces the clinical severity of this collection; no PDR isolates were identified, consistent with other Egyptian studies [44,57].

Carbapenemase production is the most clinically important mechanism of carbapenem resistance among CR-KP isolates [1,8]. Ambler class A, B and D β-lactamases predominate in *K. pneumoniae* [8,9]. The detection rate of MHT for the phenotypic assays used on the 89 CR-KP isolates was 76.4%, which is in general agreement with previous reports of 68.4% [57] and 84% [42]; mCIM was positive in 97.8% of isolates, which is consistent with other studies [42,58,59]; and the modified Carba NP test was positive in 94.4%, similar to Osama et al. [42] and Patidar et al. [60] in Egypt and India, respectively. Genotypic analysis revealed that the most common carbapenemase type was class D β-lactamases with *bla*_OXA-48_ in 94.7% of the isolates, which is in accordance with other studies performed in Egypt [17,57], Saudi Arabia [61], and Turkey [62]. *bla*_OXA-48_ is a major problem in Egypt, where it is endemic in health care settings and associated with a high plasmid burden and high transferability across antibiotic classes [63]. The conjugation data generated in the present study (discussed below) support this relationship directly.

The second most common gene was *bla*_NDM-1_ (69.1%), which agrees with previous Egyptian and international reports [18,19,64,65]. *bla*_OXA-48_ and *bla*_NDM-1_ co-occurred in 67.0% of isolates, which agrees with recent Egyptian studies that reported similarly high co-occurrence rates [17,19]. Conversely, *bla*_KPC_ was rare (5.3%), and no *bla*_IMP_ or *bla*_VIM_ were detected, in line with other regional studies [17,18,19], suggesting these genes are not common in this geographical area at present. Two CR-KP isolates were negative for all five carbapenemase genes screened, but phenotypically carbapenemase-positive, which agrees with Tawfick et al. [18]. This discrepancy may reflect the presence of carbapenemase determinants not included in the panel or the presence of other mechanisms of carbapenem resistance, such as the combined effect of ESBL production and porin loss [1,11,66]. Conversely, five other isolates were phenotypically negative but PCR-positive for one or more of the carbapenemase genes, also consistent with Tawfick et al. [18], perhaps representing silent genes or low carbapenem-hydrolyzing activity [67,68]. The same explanation plausibly applies to isolate K.104, which carried *bla*_OXA-48_ yet yielded only an intermediate mCIM result, OXA-48-type enzymes being weak carbapenemases whose activity is not always detected by phenotypic assays. The five carbapenem-susceptible isolates fall outside the concordance analysis presented in Table 5, which is restricted to the 89 carbapenem-resistant isolates on which the phenotypic assays were performed; they are reported separately here to avoid any implication that they represent additional discordance within that denominator.

It should be emphasized that the molecular characterization in this study was PCR-based, with Sanger sequencing performed on a representative subset of amplicons rather than on the entire positive collection or by whole-genome sequencing. This approach reliably confirms gene identity and variants for the sequenced isolates and is widely used in regional CR-KP surveillance [17,18,19], but it does not provide whole-genome resolution of plasmid replicon type, insertion-sequence context, or clonal lineage (e.g., ST11, ST147, ST258) for the full collection. Consequently, the term “genotype” is used here in the sense of PCR/sequencing-confirmed carbapenemase gene content rather than a comprehensive whole-genome genotype, and the plasmid carriage inferred from the conjugation and PCR data below should be regarded as functionally supported rather than physically established. Future work incorporating whole-genome sequencing and multilocus sequence typing would allow more granular assessment of clonal relatedness and plasmid architecture across this collection.

The conjugation assays performed on a representative subset of 18 CRKP isolates provide direct functional evidence bearing on this concern: carbapenemase-encoding plasmids were successfully transferred to *E. coli* J53 from 77.8% (14/18) of donors, with confirmed transfer of *bla*_NDM-1_ and *bla*_OXA-48_ (including their co-transfer from co-carriage donors) as well as *bla*_KPC_ and conferred non-susceptible meropenem, imipenem and ertapenem phenotypes in all transconjugants. This transfer efficiency indicates that, within the donor subset tested, the *bla*_NDM-1_/*bla*_OXA-48_ co-carriage genotype is frequently associated with a conjugatively competent plasmid capable of transferring carbapenem resistance into a different species. Whether this reflects clonal expansion of a small number of strains, horizontal spread of one or more plasmid lineages, or both, cannot be resolved without multilocus sequence typing and plasmid characterization, neither of which was undertaken here. The disaggregation of these outcomes by donor genotype is presented in Table 4. The transfer parameters observed here are broadly consistent with those reported for comparable carbapenemase-encoding plasmids, although direct comparison between studies is complicated by methodological heterogeneity. Working with nine OXA-48-producing *K. pneumoniae* donors of the ST147 high-risk lineage and carbapenem-susceptible *E. coli* recipients recovered from the same patients, Potel et al. obtained transconjugants from seven donors, a per-donor transfer efficiency of 77.8% that is essentially identical to the 77.8% (14/18) obtained here [69]. Reported conjugation frequencies vary more widely. Giani et al., using *E. coli* J53 as the recipient and expressing transfer per recipient cell as we do, recorded a frequency of approximately 7 × 10^−5^ for a pOXA-48a-like plasmid [70]; this lies at the lower end of the range spanned by our donors (2.1 × 10^−5^ to 6.5 × 10^−3^), although roughly fiftyfold below our mean of (3.42 ± 0.81) × 10^−3^. Such differences are to be expected, since conjugation frequency is sensitive to mating format, donor-to-recipient ratio, mating duration and the selective agents used, and frequencies expressed per donor rather than per recipient are not directly interchangeable with ours. Importantly, the efficiency with which *bla*_OXA-48_-bearing plasmids transfer has a recognized genetic basis: insertion of Tn*1999* into the *tir* gene, which encodes a transfer-inhibition protein, derepresses the conjugative machinery of the archetypal IncL/M plasmid pOXA-48a [71]. The high transfer efficiency observed in the present collection is therefore consistent with the established behavior of this plasmid family rather than exceptional for it, an observation that is itself of concern in a setting where *bla*_OXA-48_ is endemic.

The very high antimicrobial resistance observed in these isolates is likely to be multifactorial, with overlapping contributions, including the predominance of ICU-sourced isolates, where broad-spectrum antimicrobial exposure, prolonged hospitalization, invasive devices, and repeated healthcare contact are common [72]. Egyptian reports also highlighted high inpatient antimicrobial use and gaps in antimicrobial stewardship, and the need to restrict carbapenem use, as irrational use contributes directly to carbapenem resistance [73,74].

The very high level of resistance identified in this cohort should also be interpreted against the backdrop of the substantial disruption to antimicrobial stewardship that accompanied the COVID-19 pandemic and its aftermath. Multicenter surveillance from China documented that COVID-19-positive inpatients received antimicrobials considerably more often, and at higher intensity, than COVID-19-negative inpatients, frequently in the absence of a microbiologically confirmed bacterial infection, with a corresponding shift toward broad-spectrum and restricted-access agents [75]. Comparable patterns of pandemic-era antibiotic misuse, including substantial rates of non-prescribed and self-directed antibiotic use, have been reported in the general population elsewhere in the region [76], reinforcing the wider recognition of antimicrobial resistance as a growing and multifactorial threat to global public health that both predates and was plausibly amplified by the pandemic [77]. A hospital-based analysis from Slovenia similarly linked pandemic-era changes in the consumption of last-line agents, including meropenem and colistin, to a subsequent rise in the occurrence of multidrug-resistant organisms [78]. Although the isolates characterized here were collected in an Egyptian tertiary-care setting rather than during a defined pandemic surge, these regional and international observations reinforce the plausibility that broad-spectrum antibiotic overuse, whether driven by pandemic-era empirical prescribing or by pre-existing gaps in stewardship, has contributed materially to the carbapenem-resistance burden documented in this collection.

Given the scale of the resistance burden described here and the conjugative transfer demonstrated in the donor subset, therapeutic strategies that act independently of conventional β-lactam or polymyxin targets merit continued attention. Siderophore-conjugated, so-called “Trojan horse” antibiotics exploit the bacterium’s own iron-acquisition machinery to carry an antibacterial warhead across the Gram-negative outer membrane, thereby bypassing porin loss and efflux, and have shown in vitro activity against carbapenemase-producing Enterobacteriaceae, including KPC- and NDM-type producers [79]. A mechanistically related approach seeks instead to disrupt bacterial iron piracy directly, by targeting the metallophore systems on which *K. pneumoniae* and other Gram-negative pathogens depend for survival in the iron-restricted host environment [80]. Bacteriophage-based therapeutics represent a further, distinct option, offering pathogen-specific killing either alone or synergistically with residual antibiotic activity against multidrug- and extensively drug-resistant Gram-negative infections, including carbapenem-resistant *K. pneumoniae* [81]. None of these approaches is yet part of routine clinical practice, and none was evaluated against the specific *bla*_OXA-48_/*bla*_NDM-1_ co-carriage genotype characterized in this study; they are raised here to emphasize that the surveillance and stewardship measures advocated in our conclusions will need to be complemented by sustained investment in mechanistically novel therapeutics if the mobile resistance reservoir documented in these settings is to be contained.

Notably, formal statistical testing revealed no significant association between carbapenem resistance (or co-carriage of *bla*_OXA-48_/*bla*_NDM-1_) and hospital site, ward type or patient sex (Section 3.11), suggesting that resistance in this collection is widely endemic across both participating centers rather than driven by a localized outbreak or specific patient subgroup. The predominance of *bla*_OXA-48_ and *bla*_NDM-1_, together with the conjugative transferability of these plasmids, further suggests that horizontal gene transfer and healthcare-associated transmission have both contributed to resistance dissemination in these settings [19]. However, as patient-level data on prior antimicrobial exposure, hospitalization duration, invasive-device use, colonization pressure, and infection-control compliance were not collected, and as no a priori power calculation was performed for this observational surveillance study, these explanations are plausible contributing drivers rather than proven risk factors, and prospective, adequately powered, patient-level studies are required to formally demonstrate causality.

Several limitations qualify these findings. First, the conjugation experiments were performed on 18 of 94 isolates; although this subset spanned every carbapenemase genotype in the collection, the resulting transfer efficiency of 77.8% carries a wide exact confidence interval (52.4–93.6%) and should be read as an estimate derived from a small donor panel rather than as a precise property of the collection. Second, plasmid carriage was inferred from conjugation and from PCR of plasmid preparations rather than established physically; no replicon typing, plasmid sequencing or long-read genomic localization was performed, and the term “plasmid-mediated” is used here in that functional sense. Third, no multilocus sequence typing or whole-genome sequencing was undertaken, so clonal relatedness among isolates could not be assessed, and the relative contributions of clonal expansion and horizontal transfer cannot be separated. Fourth, sampling was markedly unequal between the two participating centers, and the statistical comparisons reported in Section 3.11 are exploratory, unadjusted for multiple testing, and underpowered to exclude modest associations. These constraints do not affect the descriptive prevalence data, but they do bound the mechanistic and epidemiological inferences that can be drawn from them.

## 5. Conclusions

Carbapenems are a fundamental treatment for infections caused by multidrug-resistant (MDR) *K. pneumoniae*, but the emergence of CR-KP represents a major clinical and public health challenge. In this study, all *K. pneumoniae* isolates recovered from two Egyptian tertiary care hospitals were multidrug-resistant, with 94.68% resistant to imipenem, and the carbapenem-resistant subgroup was classified as extensively drug-resistant (XDR) instead of multidrug-resistant (MDR). Colistin and tigecycline were the most active in vitro and are the main last-resort options for CR-KP infections in these settings. Carbapenemase producers were confirmed phenotypically in 98.9% and genotypically in 97.9% of isolates, with *bla*_OXA-48_ (94.7%) and *bla*_NDM-1_ (69.1%) being the predominant carbapenemase-encoding genes and co-carriage of both observed in 67.0% of isolates. Conjugation assays further showed that carbapenemase determinants were transferable to *E. coli* J53 from 77.8% of the 18 donors tested, providing functional evidence that at least part of this resistance burden is carried on conjugative plasmids. Taken together, these observations underline the importance of regular surveillance for carbapenemases in *K. pneumoniae* isolates, the molecular monitoring of conjugative transferability, and the careful stewardship of carbapenems to curb the further spread of carbapenem resistance in Egyptian healthcare facilities.

## Figures and Tables

**Table 1:** PCR primer sets used for amplification of carbapenemase-encoding genes.

Target Gene	Primer	Oligonucleotide Sequence (5′→3′)	Amplicon Size (bp)	Reference
*bla*_OXA-48_	OXA48-F OXA48-R	GCGTGGTTAAGGATGAACAC CATCAAGTTCAACCCAACCG	438	[29]
*bla*_NDM_	NDM-F NDM-R	GGTTTGGCGATCTGGTTTTC CGGAATGGCTCATCACGATC	621	[29]
*bla*_KPC_	KPC-F KPC-R	CGTCTAGTTCTGCTGTCTTG CTTGTCATCCTTGTTAGGCG	798	[29]
*bla*_IMP_	IMP-F IMP-R	GGAATAGAGTGGCTTAAYTCTC GGTTTAAYAAAACAACCACC	232	[29]
*bla*_VIM_	VIM-F VIM-R	GATGGTGTTTGGTCGCATA CGAATGCGCAGCACCAG	390	[29]

**Table 2:** Sex and age-group distribution of *K. pneumoniae* isolates recovered from hospitalized patients (*N* = 94).

Patient Characteristic	Number (%) of Isolates
Sex—Male	53 (56.4%)
Sex—Female	41 (43.6%)
Infants (<1 year)	7 (7.4%)
Children (1–14 years)	3 (3.2%)
Adults (15–64 years)	73 (77.7%)
Older adults (≥65 years)	11 (11.7%)
Age range	18 days to 75 years
Age, mean ± SD	39.58 ± 19.97 years

**Table 3:** Distribution of single and mixed carbapenemase genes among tested *K. pneumoniae* clinical isolates.

Genotype Pattern	Detected Carbapenemase Gene(s)	Number (%) of Isolates
Single carbapenemase gene	*bla*_KPC_	1 (1.1%)
	*bla*_NDM_	2 (2.1%)
	*bla*_OXA-48_	22 (23.4%)
Mixed carbapenemase genes	*bla*_KPC_ + *bla*_OXA-48_	4 (4.3%)
	*bla*_NDM_ + *bla*_OXA-48_	63 (67.0%)
Total	—	92 (97.9%)

**Table 4:** Conjugative transfer of carbapenemase-encoding plasmids from the representative CRKP donor subset (*n* = 18) to *E. coli* J53, disaggregated by donor genotype.

Donor Genotype	Donor Isolates	Donors Tested (*n*)	Donors with Confirmed Transfer (*n*)	Transfer Efficiency (%)
*bla*_OXA-48_ + *bla*_NDM-1_ (co-carriage)	K.16, K.19, K.21, K.22, K.25, K.41, K.44, K.45, K.64	9	8	88.9
*bla*_KPC_ + *bla*_OXA-48_ (co-carriage)	K.10, K.26, K.98, K.107	4	3	75.0
*bla*_OXA-48_ (single)	K.2, K.29, K.32	3	2	66.7
*bla*_NDM-1_ (single)	K.1, K.63	2	1	50.0
Overall	—	18	14	77.8 (95% CI 52.4–93.6)

Transfer efficiency is the percentage of donor strains yielding at least one confirmed transconjugant. The overall 95% confidence interval was calculated by the exact (Clopper–Pearson) binomial method; confidence intervals are not given for the individual genotype groups because of the small number of donors in each. All four *bla*_KPC_/*bla*_OXA-48_ co-carriage isolates in the collection were included as donors. The single isolate carrying *bla*_KPC_ alone (K.15) was not included, as it was carbapenem-susceptible and therefore fell outside the CRKP donor set. Conjugation frequencies, expressed as transconjugants per recipient cell, are reported in the text for the collection as a whole.

**Table 5:** Concordance between phenotypic carbapenemase detection and PCR-based genotypic detection among the 89 carbapenem-resistant *K. pneumoniae* isolates.

	Genotype Positive	Genotype Negative	Total
Phenotype positive	86	2 (K.8, K.83)	88
Phenotype indeterminate	1 (K.104)	0	1
Total	87	2	89

Phenotype positive denotes a positive result in at least one of the modified Hodge tests, mCIM or mCNP; phenotype indeterminate denotes an intermediate mCIM result with no other positive test. Concordance (phenotype positive/genotype positive) was 86/89 (96.6%). No carbapenem-resistant isolate was negative by both approaches. The phenotypic assays were applied only to the 89 carbapenem-resistant isolates; the five carbapenem-susceptible isolates are not included here.

## Data Availability

The datasets generated and analyzed during the current study are available in the article and its Supplementary Materials. Nucleotide sequences of the *bla*_OXA-48_ and *bla*_NDM-1_ genes characterized in this study have been deposited in the NCBI GenBank nucleotide sequence database under accession numbers PZ251032–PZ251036 and PZ482034–PZ482035, respectively.

## Supplementary Materials

The following supporting information can be downloaded at: https://www.mdpi.com/article/10.3390/pathogens15101043/s1, Table S1: Clinical and demographic characteristics of *K. pneumoniae* isolates; Table S2: Antimicrobial resistance, phenotypic, and genotypic profiles of the tested *K. pneumoniae* isolates; Figure S1: Singleplex PCR detection of *bla*_OXA-48_ among 12 representative *K. pneumoniae* clinical isolates (K.39–K.50); Figure S2: Multiplex PCR detection of *bla*_NDM_ and *bla*_KPC_ among 14 representative *K. pneumoniae* clinical isolates (K.6–K.21).

## Abbreviations

The following abbreviations are used in this manuscript:

Abbreviation	Definition
AMR	Antimicrobial resistance
CFU	Colony-forming unit(s)
CLSI	Clinical and Laboratory Standards Institute
CRE	Carbapenem-resistant Enterobacteriaceae
CR-KP	Carbapenem-resistant *Klebsiella pneumoniae*
CTAB	Cetyltrimethylammonium bromide
ESBL	Extended-spectrum β-lactamase
EUCAST	European Committee on Antimicrobial Susceptibility Testing
ICU	Intensive care unit
IMP	Imipenemase
KPC	*Klebsiella pneumoniae* carbapenemase
LB	Luria-Bertani (broth/agar)
MBL	Metallo-β-lactamase
mCIM	Modified carbapenem inactivation method
mCNP	Modified Carba NP test
MDR	Multidrug-resistant
MHT	Modified Hodge test
MIC	Minimum inhibitory concentration
NDM	New Delhi metallo-β-lactamase
OXA-48	Oxacillinase-48
PBS	Phosphate-buffered saline
PDR	Pandrug-resistant
TAE	Tris-acetate-EDTA (buffer)
T/R ratio	Transconjugant-to-recipient CFU ratio
VIM	Verona integron-encoded metallo-β-lactamase
XDR	Extensively drug-resistant
