# Supplementary material for: Molecular Epidemiology and Conjugative Transferability of blaOXA-48/blaNDM-1 Co-Occurrence in Extensively Drug-Resistant Klebsiella pneumoniae from Egyptian Tertiary Care Hospitals
Source: Pathogens. 2026 Oct 2;15(10):1043. doi: 10.3390/pathogens15101043 (PMC13708560; doi:10.3390/pathogens15101043)
Supplement: Supplementary file 1 [file pathogens-15-01043-s001.zip › pathogens-4550487-supplementary.pdf]

Supplementary Materials

Molecular Epidemiology and **Conjugative** Transferability of *bla*$_{OXA-48}$/*bla*$_{NDM-1}$ Co-Occurrence in Extensively Drug-Resistant *Klebsiella pneumoniae* from Egyptian Tertiary Care Hospitals

Table S1. Clinical and demographic characteristics of *K. pneumoniae* isolates recovered from hospitalized patients, including specimen source, ward type, patient sex and age, and hospital site.

Isolate No.	Specimen Source	Ward Type	Sex/Age (Y)	Hospital Name
K.1	Blood	ICU	M/5	El-Demerdash Hospital
K.2	Urine	Infant incubator unit	F/25 Days	El-Demerdash Hospital
K.4	Urine	ICU	M/33	El-Demerdash Hospital
K.6	Wound	Burns unit	M/35	El-Demerdash Hospital
K.7	Catheter	ICU	M/32	El-Demerdash Hospital
K.8	Urine	Gastroenterology unit	M/31	El-Demerdash Hospital
K.9	Blood	Burns unit	M/23	El-Demerdash Hospital
K.10	Blood	ICU	M/53	El-Demerdash Hospital
K.11	Blood	ICU	M/65	El-Demerdash Hospital
K.12	Aspirate	Surgical unit	M/33	El-Demerdash Hospital
K.14	Endotracheal tube	ICU	M/32	El-Demerdash Hospital
K.15	Urine	Urology unit	M/56	El-Demerdash Hospital
K.16	Blood	ICU	M/14	El-Demerdash Hospital
K.17	Endotracheal tube	ENT unit	M/53	El-Demerdash Hospital
K.18	Blood	Burns unit	M/68	El-Demerdash Hospital
K.19	Urine	Surgical unit	F/33	El-Demerdash Hospital
K.20	Wound	Burns unit	F/48	El-Demerdash Hospital
K.21	Blood	Surgical unit	F/35	El-Demerdash Hospital
K.22	Sputum	ICU	F/68	El-Demerdash Hospital
K.23	Endotracheal tube	ICU	F/21	El-Demerdash Hospital
K.24	Urine	ICU	F/18	El-Demerdash Hospital
K.25	Wound	Surgical unit	M/33	El-Demerdash Hospital
K.26	Blood	Surgical unit	F/37	El-Demerdash Hospital
K.27	Urine	Urology unit	M/42	El-Demerdash Hospital
K.28	Sputum	Gastroenterology unit	M/25	El-Demerdash Hospital
K.29	Blood	ICU	F/16	El-Demerdash Hospital
K.30	Blood	Cardiology unit	F/35	El-Demerdash Hospital
K.31	Endotracheal tube	Gastroenterology unit	M/21	El-Demerdash Hospital
K.32	Urine	ICU	M/38	El-Demerdash Hospital
K.33	Blood	ICU	F/43	El-Demerdash Hospital
K.34	Urine	Urology unit	M/54	El-Demerdash Hospital

Isolate No.	Specimen Source	Ward Type	Sex/Age (Y)	Hospital Name
K.35	Blood	ICU	M/29	El-Demerdash Hospital
K.36	Sputum	ICU	F/57	El-Demerdash Hospital
K.37	Sputum	ICU	F/19	El-Demerdash Hospital
K.38	Blood	Infant incubator unit	F/20 Days	El-Demerdash Hospital
K.39	Urine	Gastroenterology unit	F/60	El-Demerdash Hospital
K.40	Urine	ICU	F/75	El-Demerdash Hospital
K.41	Wound	ICU	M/50	El-Demerdash Hospital
K.42	Endotracheal tube	Infant incubator unit	F/26 Days	El-Demerdash Hospital
K.43	Blood	ICU	F/63	El-Demerdash Hospital
K.44	Blood	ICU	M/44	El-Demerdash Hospital
K.45	Urine	Urology unit	M/39	El-Demerdash Hospital
K.46	Blood	Infant incubator unit	F/18 Days	El-Demerdash Hospital
K.47	Endotracheal tube	Infant incubator unit	M/22 Days	El-Demerdash Hospital
K.48	Blood	ICU	M/51	El-Demerdash Hospital
K.49	Urine	Infant incubator unit	F/27 Days	El-Demerdash Hospital
K.50	Blood	Infant incubator unit	F/21 Days	El-Demerdash Hospital
K.51	Endotracheal tube	ICU	M/63	El-Demerdash Hospital
K.52	Urine	Urology unit	M/68	El-Demerdash Hospital
K.53	Pus	ICU	M/29	El-Demerdash Hospital
K.54	Sputum	ENT unit	F/48	El-Demerdash Hospital
K.55	Pus	Surgical unit	M/25	El-Demerdash Hospital
K.56	Urine	Surgical unit	M/42	El-Demerdash Hospital
K.57	Sputum	ICU	M/23	El-Demerdash Hospital
K.58	Blood	ICU	M/65	El-Demerdash Hospital
K.60	Endotracheal tube	ENT unit	F/35	El-Demerdash Hospital
K.61	Pus	ICU	M/32	El-Demerdash Hospital
K.62	Pus	Neurology unit	M/62	El-Demerdash Hospital
K.63	Sputum	ICU	F/75	El-Demerdash Hospital
K.64	Pus	Surgical unit	M/44	El-Demerdash Hospital
K.65	Wound	Surgical unit	M/56	El-Demerdash Hospital
K.66	Urine	Urology unit	F/35	El-Demerdash Hospital
K.67	Blood	ICU	M/56	El-Demerdash Hospital
K.68	Blood	ICU	M/29	El-Demerdash Hospital
K.69	Endotracheal tube	ICU	F/48	El-Demerdash Hospital
K.70	Sputum	ICU	F/63	El-Demerdash Hospital
K.71	Pus	ICU	F/37	El-Demerdash Hospital
K.72	Blood	ICU	F/72	El-Demerdash Hospital
K.73	Blood	ICU	F/35	El-Demerdash Hospital

Isolate No.	Specimen Source	Ward Type	Sex/Age (Y)	Hospital Name
K.74	Urine	ICU	M/40	El-Demerdash Hospital
K.75	Urine	Surgical unit	M/19	El-Demerdash Hospital
K.76	Pus	Surgical unit	F/55	El-Demerdash Hospital
K.77	Urine	Urology unit	M/68	El-Demerdash Hospital
K.78	Pus	ICU	F/66	El-Demerdash Hospital
K.81	Blood	ICU	F/60	El-Demerdash Hospital
K.82	Urine	Surgical unit	M/29	El-Demerdash Hospital
K.83	Blood	ICU	M/53	El-Demerdash Hospital
K.85	Sputum	ENT unit	M/32	El-Demerdash Hospital
K.88	Urine	Urology unit	F/19	El-Demerdash Hospital
K.89	Blood	ENT unit	M/31	El-Demerdash Hospital
K.90	Urine	ICU	F/75	Mabart El-Asafra Hospital
K.91	Urine	Urology unit	F/19	Mabart El-Asafra Hospital
K.92	Blood	Surgical unit	F/61	Mabart El-Asafra Hospital
K.93	Blood	ENT unit	M/31	Mabart El-Asafra Hospital
K.94	Urine	Urology unit	M/23	Mabart El-Asafra Hospital
K.96	Sputum	ENT unit	M/44	Mabart El-Asafra Hospital
K.97	Urine	Surgical unit	M/14	Mabart El-Asafra Hospital
K.98	Blood	ICU	F/63	Mabart El-Asafra Hospital
K.101	Blood	ICU	M/62	Mabart El-Asafra Hospital
K.104	Pus	ICU	F/37	Mabart El-Asafra Hospital
K.105	Sputum	ICU	F/60	Mabart El-Asafra Hospital
K.107	Endotracheal tube	ICU	M/42	Mabart El-Asafra Hospital
K.108	Urine	Neurology unit	F/35	Mabart El-Asafra Hospital
K.109	Urine	Urology unit	M/53	Mabart El-Asafra Hospital

Table S2. Antimicrobial resistance, phenotypic, and genotypic profiles of the tested *K. pneumoniae* clinical isolates.

Isolate	AMR Profile	Positive Phenotypic Test(s)	Detected Carbapenemase Gene(s)
K.1	MDR + CR	MHT, mCIM*	bla_{NDM-1}
K.2	MDR + CR	MHT, mCIM, mCNP	bla_{OXA-48}
K.4	MDR + CR	MHT, mCIM, mCNP	bla_{OXA-48}, bla_{NDM-1}
K.6	MDR + CR	mCIM, mCNP	bla_{OXA-48}, bla_{NDM-1}
K.7	MDR + CS	-	bla_{OXA-48}, bla_{NDM-1}
K.8	MDR + CR	mCIM, mCNP	-
K.9	MDR + CR	MHT, mCIM, mCNP	bla_{OXA-48}, bla_{NDM-1}

Isolate	AMR Profile	Positive Phenotypic Test(s)	Detected Carbapenemase Gene(s)
K.10	MDR + CR	MHT, mCIM, mCNP	*bla*$_{OXA-48}$, *bla*$_{KPC}$
K.11	MDR + CR	MHT, mCIM, mCNP	*bla*$_{OXA-48}$, *bla*$_{NDM-1}$
K.12	MDR + CR	MHT, mCIM, mCNP	*bla*$_{OXA-48}$, *bla*$_{NDM-1}$
K.14	MDR + CR	MHT, mCIM, mCNP	*bla*$_{OXA-48}$, *bla*$_{NDM-1}$
K.15	MDR + CS	-	*bla*$_{KPC}$
K.16	MDR + CR	MHT, mCIM, mCNP	*bla*$_{OXA-48}$, *bla*$_{NDM-1}$
K.17	MDR + CR	MHT, mCIM, mCNP	*bla*$_{OXA-48}$, *bla*$_{NDM-1}$
K.18	MDR + CR	MHT, mCIM, mCNP	*bla*$_{OXA-48}$
K.19	MDR + CR	MHT, mCIM, mCNP	*bla*$_{OXA-48}$, *bla*$_{NDM-1}$
K.20	MDR + CS	-	*bla*$_{OXA-48}$
K.21	MDR + CR	MHT, mCIM, mCNP	*bla*$_{OXA-48}$, *bla*$_{NDM-1}$
K.22	MDR + CR	MHT, mCIM, mCNP	*bla*$_{OXA-48}$, *bla*$_{NDM-1}$
K.23	MDR + CR	MHT, mCIM, mCNP	*bla*$_{OXA-48}$
K.24	MDR + CR	mCIM, mCNP	*bla*$_{OXA-48}$, *bla*$_{NDM-1}$
K.25	MDR + CR	MHT, mCIM, mCNP	*bla*$_{OXA-48}$, *bla*$_{NDM-1}$
K.26	MDR + CR	MHT, mCIM, mCNP	*bla*$_{OXA-48}$, *bla*$_{KPC}$
K.27	MDR + CR	mCIM, mCNP	*bla*$_{OXA-48}$, *bla*$_{NDM-1}$
K.28	MDR + CR	MHT, mCIM, mCNP	*bla*$_{OXA-48}$, *bla*$_{NDM-1}$
K.29	MDR + CR	MHT, mCIM, mCNP	*bla*$_{OXA-48}$
K.30	MDR + CR	MHT, mCIM, mCNP	*bla*$_{OXA-48}$, *bla*$_{NDM-1}$
K.31	MDR + CR	MHT, mCIM, mCNP	*bla*$_{OXA-48}$, *bla*$_{NDM-1}$
K.32	MDR + CR	MHT, mCIM, mCNP	*bla*$_{OXA-48}$
K.33	MDR + CR	MHT, mCIM, mCNP	*bla*$_{OXA-48}$
K.34	MDR + CR	MHT, mCIM, mCNP	*bla*$_{OXA-48}$, *bla*$_{NDM-1}$
K.35	MDR + CR	MHT, mCIM, mCNP	*bla*$_{OXA-48}$, *bla*$_{NDM-1}$
K.36	MDR + CR	MHT, mCIM, mCNP	*bla*$_{OXA-48}$, *bla*$_{NDM-1}$
K.37	MDR + CR	MHT, mCIM, mCNP	*bla*$_{OXA-48}$
K.38	MDR + CR	MHT, mCIM, mCNP	*bla*$_{OXA-48}$, *bla*$_{NDM-1}$
K.39	MDR + CR	MHT, mCIM, mCNP	*bla*$_{OXA-48}$, *bla*$_{NDM-1}$
K.40	MDR + CR	MHT, mCIM, mCNP	*bla*$_{OXA-48}$, *bla*$_{NDM-1}$
K.41	MDR + CR	MHT, mCIM, mCNP	*bla*$_{OXA-48}$, *bla*$_{NDM-1}$
K.42	MDR + CR	mCIM, mCNP	*bla*$_{OXA-48}$, *bla*$_{NDM-1}$
K.43	MDR + CR	MHT, mCIM, mCNP	*bla*$_{OXA-48}$, *bla*$_{NDM-1}$
K.44	MDR + CR	MHT, mCIM, mCNP	*bla*$_{OXA-48}$, *bla*$_{NDM-1}$
K.45	MDR + CR	MHT, mCIM, mCNP	*bla*$_{OXA-48}$, *bla*$_{NDM-1}$
K.46	MDR + CR	MHT, mCIM	*bla*$_{OXA-48}$, *bla*$_{NDM-1}$
K.47	MDR + CR	MHT, mCIM, mCNP	*bla*$_{OXA-48}$
K.48	MDR + CR	MHT, mCIM, mCNP	*bla*$_{OXA-48}$

Isolate	AMR Profile	Positive Phenotypic Test(s)	Detected Carbapenemase Gene(s)
K.49	MDR + CR	MHT, mCIM, mCNP	*bla*$_{OXA-48}$
K.50	MDR + CR	mCIM, mCNP	*bla*$_{OXA-48}$, *bla*$_{NDM-1}$
K.51	MDR + CR	mCIM, mCNP	*bla*$_{OXA-48}$, *bla*$_{NDM-1}$
K.52	MDR + CR	MHT, mCIM, mCNP	*bla*$_{OXA-48}$, *bla*$_{NDM-1}$
K.53	MDR + CR	MHT, mCIM, mCNP	*bla*$_{OXA-48}$, *bla*$_{NDM-1}$
K.54	MDR + CR	mCIM, mCNP	*bla*$_{OXA-48}$
K.55	MDR + CR	MHT, mCIM, mCNP	*bla*$_{OXA-48}$
K.56	MDR + CR	mCIM, mCNP	*bla*$_{OXA-48}$, *bla*$_{NDM-1}$
K.57	MDR + CR	mCIM, mCNP	*bla*$_{OXA-48}$, *bla*$_{NDM-1}$
K.58	MDR + CR	mCIM, mCNP	*bla*$_{OXA-48}$, *bla*$_{NDM-1}$
K.60	MDR + CR	mCIM, mCNP	*bla*$_{OXA-48}$, *bla*$_{NDM-1}$
K.61	MDR + CR	mCIM, mCNP	*bla*$_{OXA-48}$, *bla*$_{NDM-1}$
K.62	MDR + CR	MHT, mCIM, mCNP	*bla*$_{OXA-48}$, *bla*$_{NDM-1}$
K.63	MDR + CR	MHT, mCIM, mCNP	*bla*$_{NDM-1}$
K.64	MDR + CR	MHT, mCIM	*bla*$_{OXA-48}$, *bla*$_{NDM-1}$
K.65	MDR + CR	MHT, mCIM, mCNP	*bla*$_{OXA-48}$, *bla*$_{NDM-1}$
K.66	MDR + CR	MHT, mCIM, mCNP	*bla*$_{OXA-48}$, *bla*$_{NDM-1}$
K.67	MDR + CR	MHT, mCIM, mCNP	*bla*$_{OXA-48}$, *bla*$_{NDM-1}$
K.68	MDR + CR	MHT, mCIM, mCNP	*bla*$_{OXA-48}$, *bla*$_{NDM-1}$
K.69	MDR + CR	mCIM, mCNP	*bla*$_{OXA-48}$, *bla*$_{NDM-1}$
K.70	MDR + CR	MHT, mCIM, mCNP	*bla*$_{OXA-48}$, *bla*$_{NDM-1}$
K.71	MDR + CR	MHT, mCIM, mCNP	*bla*$_{OXA-48}$
K.72	MDR + CR	MHT, mCIM, mCNP	*bla*$_{OXA-48}$
K.73	MDR + CR	MHT, mCIM, mCNP	*bla*$_{OXA-48}$, *bla*$_{NDM-1}$
K.74	MDR + CR	MHT, mCIM, mCNP	*bla*$_{OXA-48}$
K.75	MDR + CR	MHT, mCIM, mCNP	*bla*$_{OXA-48}$
K.76	MDR + CR	MHT, mCIM, mCNP	*bla*$_{OXA-48}$, *bla*$_{NDM-1}$
K.77	MDR + CR	mCIM, mCNP	*bla*$_{OXA-48}$, *bla*$_{NDM-1}$
K.78	MDR + CR	MHT, mCIM, mCNP	*bla*$_{OXA-48}$, *bla*$_{NDM-1}$
K.81	MDR + CR	mCIM, mCNP	*bla*$_{OXA-48}$, *bla*$_{NDM-1}$
K.82	MDR + CR	MHT, mCIM, mCNP	*bla*$_{OXA-48}$, *bla*$_{NDM-1}$
K.83	MDR + CR	MHT, mCIM, mCNP	-
K.85	MDR + CR	MHT, mCIM, mCNP	*bla*$_{OXA-48}$, *bla*$_{NDM-1}$
K.88	MDR + CR	MHT, mCIM, mCNP	*bla*$_{OXA-48}$
K.89	MDR + CR	mCIM, mCNP	*bla*$_{OXA-48}$, *bla*$_{NDM-1}$
K.90	MDR + CR	MHT, mCIM, mCNP	*bla*$_{OXA-48}$
K.91	MDR + CR	mCIM, mCNP	*bla*$_{OXA-48}$, *bla*$_{NDM-1}$
K.92	MDR + CS	-	*bla*$_{OXA-48}$

Isolate	AMR Profile	Positive Phenotypic Test(s)	Detected Carbapenemase Gene(s)
K.93	MDR + CR	MHT, mCIM, mCNP	bla_{OXA-48}, bla_{NDM-1}
K.94	MDR + CR	mCIM, mCNP	bla_{OXA-48}, bla_{NDM-1}
K.96	MDR + CR	mCIM	bla_{OXA-48}, bla_{NDM-1}
K.97	MDR + CR	MHT, mCIM, mCNP	bla_{OXA-48}, bla_{NDM-1}
K.98	MDR + CR	MHT, mCIM, mCNP	bla_{OXA-48}, bla_{KPC}
K.101	MDR + CR	MHT, mCIM, mCNP	bla_{OXA-48}, bla_{NDM-1}
K.104	MDR + CR	mCIM*	bla_{OXA-48}
K.105	MDR + CR	MHT, mCIM, mCNP	bla_{OXA-48}, bla_{NDM-1}
K.107	MDR + CR	MHT, mCIM, mCNP	bla_{OXA-48}, bla_{KPC}
K.108	MDR + CS	-	bla_{OXA-48}
K.109	MDR + CR	MHT, mCIM, mCNP	bla_{OXA-48}, bla_{NDM-1}

* mCIM* indicates intermediate results with mCIM.

MDR, multidrug-resistant; CR, carbapenem-resistant; CS, carbapenem-sensitive.

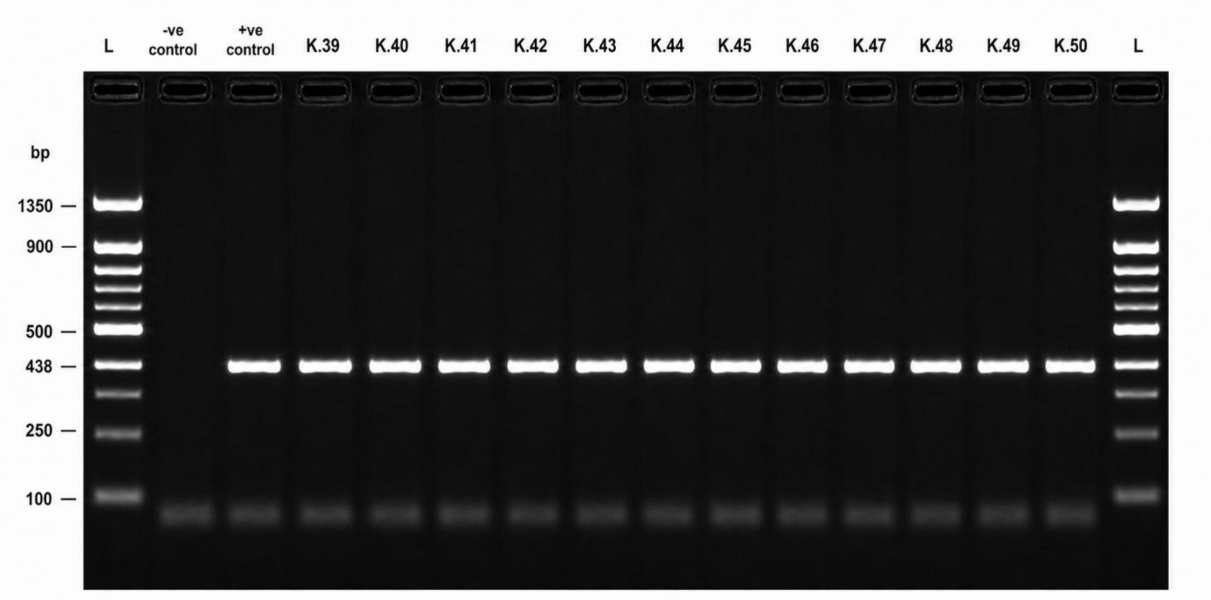

Figure S1. Singleplex PCR detection of bla_{OXA-48} among 12 representative *K. pneumoniae* clinical isolates (K.39–K.50). Lane L, 50 bp DNA ladder; –ve control, no-template negative control (nuclease-free water); +ve control, *Klebsiella pneumoniae* NCTC 13442 (bla_{OXA-48}-positive reference strain, 438 bp). An amplicon size of 438 bp indicates positive amplification of bla_{OXA-48}; all isolates shown were positive.

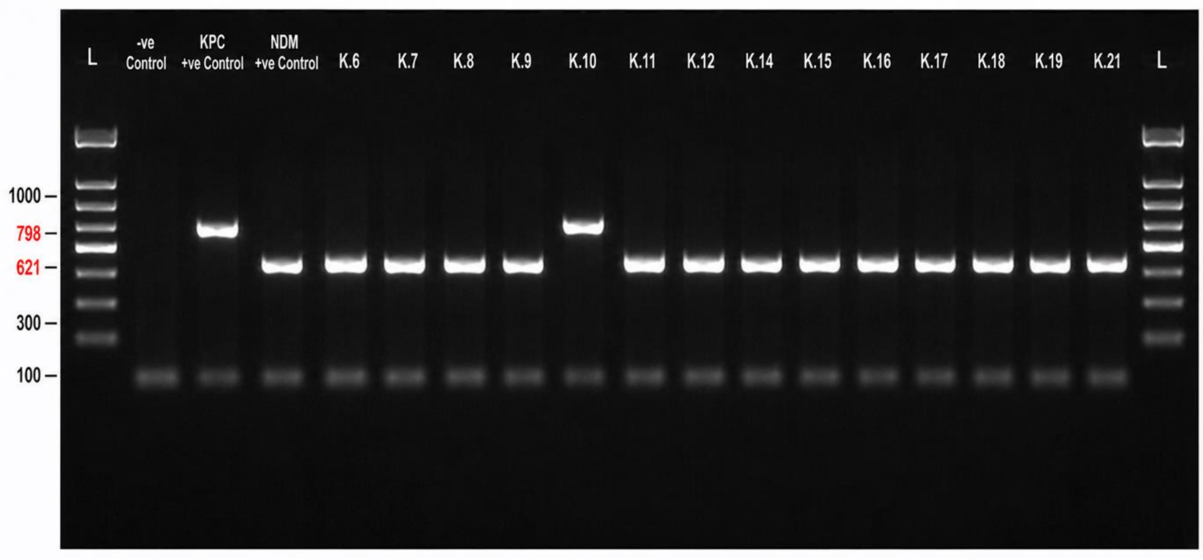

Figure S2. Multiplex PCR detection of *bla*$_{NDM}$ and *bla*$_{KPC}$ among 14 representative *K. pneumoniae* clinical isolates (K.6–K.21). Lane L, 100 bp DNA ladder; –ve control, no-template negative control (nuclease-free water); KPC +ve control, *Klebsiella pneumoniae* ATCC® BAA-1705 (*bla*$_{KPC}$-positive reference strain, 798 bp); NDM +ve control, *Klebsiella pneumoniae* ATCC® BAA-2146 (*bla*$_{NDM}$ -positive reference strain, 621 bp). Amplicon sizes of 798 bp and 621 bp indicate positive amplification of *bla*$_{KPC}$ and *bla*$_{NDM}$, respectively; isolate K.10 shows the 798 bp *bla*$_{KPC}$-positive band, while all other isolates shown display the 621 bp *bla*$_{NDM}$-positive band.
